# Evaluating the Resilience of Drinking Water Systems to Severe Weather Events Using Boil Water Notices and Bottled Water Sales

**DOI:** 10.1021/acs.est.5c18579

**Published:** 2026-07-31

**Authors:** Marlee Shaffer, Nora Awad, Frances Davenport, Sarah Fakhreddine

**Affiliations:** † Department of Civil and Environmental Engineering, 6612 Carnegie Mellon University, Pittsburgh, Pennsylvania 15213, United States; ‡ Department of Civil and Environmental Engineering, 3447 Colorado State University, Fort Collins, Colorado 80523, United States

**Keywords:** water quality, drinking water systems, severe weather, boil water notices, resilience

## Abstract

Increasing weather-driven disruptions to water supplies have raised growing concerns about the resilience of drinking water infrastructure and equitable access to safe drinking water during severe weather events. This study seeks to address gaps in evaluating and understanding the resilience of community water systems (CWSs) to severe weather events at regional scales, including both the physical infrastructure and population served. We developed an approach that combines boil water notice (BWN) occurrence and duration as a metric of CWS performance and bottled water sales to capture community response to water disruptions. We conducted multiple fixed-effects panel regressions to quantify relationships of these metrics to severe weather events and operational and sociodemographic characteristics using a statewide analysis of Texas, United States from 2010 to 2022. We found that small, publicly owned, and groundwater-sourced CWSs are most susceptible to BWNs. Winter weather and hurricanes significantly increased BWN duration by 1.6 and 1.1 days, respectively. During weather-driven BWNs, high-income counties had a significant increase in bottled water purchasing (+30.9%) while low-income counties did not exhibit a significant increase. This study provides a better understanding of differences in vulnerability of CWSs to weather events and the associated response of populations served at regional scales.

## Graphical abstract

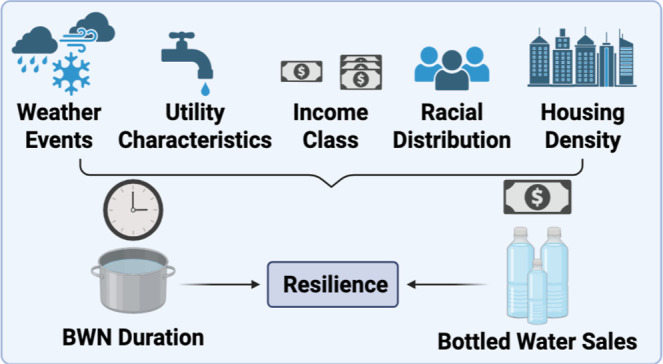

## 1 Introduction

Increasing frequency and intensity of severe-weather events globally have raised concerns about the resilience of drinking water infrastructure and potential impacts to public health.^1–3^ Notable recent examples include Winter Storm Uri in February 2021, which left more than 16 million people in Texas, United States, under boil water notices.^4,5^ Similarly, in August 2022, severe flooding in Jackson, Mississippi, United States, exacerbated existing water system issues, resulting in prolonged water service disruptions, widespread boil water notices across the city, and difficulties finding alternative water sources.^6^ These drinking water system failures have prompted growing calls to enhance water system resilience to severe weather events.^7^

The resilience of a drinking water system is broadly defined as its ability to withstand and quickly recover from a failure or disruption.^7,8^ In evaluating water system resilience, previous studies have emphasized the importance of considering water infrastructure systems as sociotechnical systems owing to the close interaction of consumers with their water supply.^3,9–12^ The five dimensions of resilience spanning economic, environmental, governance, social, and infrastructure highlight the interconnectedness of consumers and water infrastructure and complexity of assessing drinking water system resilience.^13^

While there is no single metric for drinking water system resilience, boil water notices (BWNs) can serve as a key performance-based metric to evaluate the resilience of drinking water systems to severe weather events. A drinking water system (i.e., utility or community water system (CWS)) is required to issue a BWN (or boil water advisory) when an event occurs that may degrade water quality. BWNs are also precautionarily issued for low distribution pressure, water outages, inadequate disinfectant residuals, or infrastructure concerns to safeguard public health.^14–16^ Between 2012 and 2014, over 20,000 BWNs were reported across the United States, with half resulting from system breaks and a third from low distribution pressure or confirmed microbial contamination.^17^ In 2021 alone, an analysis of BWNs issued across 20 states found 79.8% attributed to system breaks, repairs, or pressure loss, which can all lead to microbial contamination.^14^ The vulnerability of a water system to a BWN may vary depending on the operational characteristics of the system. For example, for very small systems serving fewer than 500 people, microbial contamination was the leading cause of BWNs, accounting for 43.2% of notices.^14^ Severe weather events and aging infrastructure have increased the frequency of BWNs nationwide.^18^ Infrastructure failures may also result from high temperatures, increased water demand, seismic activity, and changes in soil moisture, leading to an increase in BWNs.^19–23^ Elevated temperatures can reduce chlorine residuals, increasing the risk of microbial contamination or high turbidity levels, further compromising water quality.^20,22,24–26^

Despite the importance of understanding the impacts of severe weather on BWNs, it is difficult to evaluate these processes at large, regional scales that capture various weather conditions and water system characteristics. Unlike Safe Drinking Water Act (SDWA) violations, BWNs are not tracked in a centralized national database, despite thousands of notices issued annually in the United States.^14^ Regional studies on BWNs for the United States have assessed summary statistics on number, duration, and cause in Kentucky and West Virginia; however, they do not correlate BWNs with severe weather events.^14,18^ Furthermore, at the national level, holistic reports face large data gaps, which may impact overall study results.^14^ Other work has assessed infrastructure vulnerabilities or examined demographic disparities in response to specific severe weather events, like Winter Storm Uri.^5,27^ Furthermore, smaller-scale severe weather events are important for CWS resilience and are often overlooked compared to extreme events such as hurricanes or severe winter weather.^28^ The lack of standardized reporting on BWNs limits our understanding of their frequency, causes, and impacts, as well as efforts to evaluate resilience across water systems.

Similarly, understanding community response to BWNs provides valuable insights into the resilience of communities themselves and vulnerable populations. The ability of a community to comply with a BWN may vary with sociodemographic characteristics, such as rurality or income, potentially exacerbating environmental and social inequities.^3^ The specific type of averting action may also be impacted by severe weather. For example, purchasing bottled water may be the only viable option if concurrent electrical or gas outages prevent boiling water. Previous studies have examined public responses to BWNs primarily through surveys, focusing on risk communication and compliance.^29^ −^30,31^ However, despite the prevalence of BWNs, few studies have quantitatively evaluated how severe weather, operational system characteristics, and community sociodemographics influence BWN outcomes (e.g., duration) and community response (e.g., averting actions).

Accordingly, this study seeks to address key knowledge gaps in how severe weather events have impacted the duration of BWNs and community response during a BWN. Our primary objective is to better understand the resilience of drinking water systems and how subsequent community response varies based on the type of severe weather event, water system characteristics, and community sociodemographics. BWN occurrence and duration are used to understand how drinking water systems withstand and rebound from weather-related disruptions. We use bottled water sales as a proxy for community response due to the availability of sales data at large scales and the importance of alternative water supply during severe weather events. To our knowledge, this is the first regional study on how severe weather events impact BWN duration and subsequent bottled water sales as a proxy for community response. We focus on Texas as a case study, given its high number of drinking water systems, data availability, and range of severe weather events. Our findings highlight key vulnerabilities within water systems and offer a transferable methodology for assessing BWN trends and resilience of drinking water systems.

## 2 Materials and Methods

### 2.1 Study Area

Texas has the largest number of active community water systems (CWSs; 4,673) in the United States^32^ with publicly available records of BWN issuance. The EPA defines a CWS as a public water system that supplies water to at least 15 connections used by year-round residents.^14^ The distribution of CWS characteristics in Texas shows 62% are publicly owned, 28% are surface water sourced, 76% are small, and 24% are medium or large (Table 1). This is similar to the national distribution of CWSs where 53% are publicly owned, 24% are surface water sourced, 81% are small, and 19% are medium or large.^33^ We filtered for active CWSs and used the same CWSs for every year.Therefore, any systems that became inactive during the study period were excluded.^14^ Texas experiences various types of weather events, including hurricanes, tornadoes, winter storms, and droughts, further highlighting its value as a case study for resilience (Table S1). During the study period, Texas experienced extreme weather events, including Winter Storm Uri in February of 2021, Hurricane Harvey in 2017, and Hurricane Laura in 2020. Additionally, given the size of the state, CWSs capture a range of operational (e.g., system size, ownership, source) and sociodemographic characteristics (e.g., rurality, racial distribution), enabling analyses of potential interactions of these variables on BWN duration and community response.

**Table 1:** Summary of CWS characteristics^a^

		Texas CWS database	occurrence model	duration model
		(*n* = 4,673)	(*n* = 3,114)	(*n* = 3,078)
size	small	3,545 (75.9%)	2,384 (76.6%)	2,353 (76.4%)
	medium/large	1,128 (24.1%)	730 (23.4%)	725 (23.5%)
owner	private	1,749 (38.4%)	1,242 (39.9%)	1,223 (39.7%)
	public	2,879 (61.6%)	1,872 (60.1%)	1,855 (60.3%)
source	groundwater	3,344 (71.6%)	2,169 (69.7%)	2,138 (69.5%)
	surface water	1,329 (28.4%)	945 (30.3%)	940 (30.5%)

a The data show the number of CWSs that fall into the specific category followed by the percentage of total CWSs in the analysis in the category in parentheses. Groundwater under the influence of surface water (*n* = 24, 0.5%) was included with groundwater for the source category.

### 2.2 Data Acquisition and Processing

#### 2.2.1 Boil Water Notice Data

Statewide data on BWNs issued from 2010 to 2022 were obtained from the Texas Commission on Environmental Quality. BWNs were issued for six categories of causes in the data set: low distribution pressure, low disinfectant residual, microbial contamination, turbidity, water outage, and “other”. The “other” designation signifies that the cause did not fall within the five main categories and may include equipment failures, construction, and odor, but is not specific. When biological contaminants, such as *E. coli*, or physicochemical parameters, such as turbidity, exceed the maximum limits, CWSs are required to issue a boil water notice (BWN) within 24 h.^18^ Each BWN record included the issue and rescind date, CWS identifier, operational information (e.g., source water, connections, population served), and reported cause. After filtering for active CWSs, the final occurrence model (Section 2.3) data set included 18,839 BWNs issued by 3,114 CWSs, representing 66% of the total CWSs in Texas (4,673, Table 1). The duration of each BWN was calculated using the issuance data and rescind date. The final duration model (Section 2.4) data set included 18,276 BWNs issued by 3,078 CWSs. We used winsorization to cap extreme outliers at the 95th percentile of our data (17 days) to account for outlier presence while producing a robust model. All descriptive statistics are reported using the raw duration model data set (Table S3).

#### 2.2.2 Severe Weather Event Data

A BWN was classified as weather-related if it occurred during or within 3 days following a reported severe weather event. The National Oceanic and Atmospheric Administration (NOAA) Storm Events Database^34^ was used to identify the occurrence of severe weather events based on the county or zone affected and the date the weather event started. NOAA severe weather event categories were aggregated into six descriptive categories: heat or drought, winter weather, hurricanes, tornadoes, floods, or storms (Table S1). CWS service boundaries were obtained from the Texas Water Development Board.^35^ If more than 10% of the CWS service area was within a county or NWS zone where a severe weather event occurred, the severe weather event was assigned to the BWN entry. The threshold was selected by conducting a sensitivity analysis across thresholds ranging from 1% to 50%, which revealed largely stable CWS geographic coverage, with fewer than 2.42% of CWSs losing assignment at any threshold (Figure S1). The 10% threshold was selected to exclude artifacts from boundary misalignments.

#### 2.2.3 Operational and Sociodemographic Data

Additional sources provided relevant operational and sociodemographic data (Table S2). Operational characteristics of CWSs (service connection count, ownership type, and water source) were obtained from the EPA Safe Drinking Water Information System database.^36^ Utility size was based on the population served following EPA classifications. Very small (<500) and small (501-3,300) systems were combined into a single small category (≤3,300), and systems serving >3,300 people were classified as medium/large.

Sociodemographic data were available at the county level for each year in our analysis. Yearly county-level estimates of inflation-adjusted median household income and percent below the poverty line were sourced from the US Census Bureau Small Area Income and Poverty Estimates program.^37^ We used income thresholds defined by the Community Reinvestment Act (low to moderate income: <80% area median income (AMI), middle income: 80% to 120% AMI, high income: >120% AMI).^38^ County population, number of housing units, and population by race were obtained from the US Census Bureau Population and Housing Unit Estimates Program for 2010 to 2022.^39^ Racial distribution was represented by the percent of the population identifying as Black, Hispanic, Asian, or Other. Counties were classified as majority people of color if >50% of residents identified as Black, Asian, and Hispanic or Other. Housing density was calculated by dividing the number of housing units by the county land area obtained from the Texas Counties Cartographic Boundary Map.^40^ Urban communities were defined by housing density ≥64 units per square mile, and rural communities had <64 units per square mile.^41^

For the 204 CWSs that spanned multiple counties, sociodemographic variables were calculated using an area-weighted average of the county-level data. Area-weighted averaging assumes uniform demographic distribution within counties, which may introduce measurement error for systems whose service areas capture nonrepresentative portions of a county’s population. While finer spatial resolutions (e.g., census tract) are available through the ACS 5-year estimates, yearly estimates were only available at the county level. Given that our panel regression requires annual demographic data to capture year-to-year variation, the 1-year data were used as recommended by ACS.^42^

#### 2.2.4 Bottled Water Sales Data

Weekly bottled water sales data from January 2010 to December 2020 (the most recent data available at the time of this study) were obtained from the NielsenIQ dataset, which covers sales from 90 retail chains including grocery, drug, convenience, and mass merchandise stores. This dataset represents over 50% of US grocery and drug store sales and over 30% of mass merchandiser sales.^43^ Weekly sales (in dollars and ounces) were reported for over 5,000 bottled water products. Sales were aggregated to the county level as the data are deidentified at the store level in the NielsenIQ database, precluding analysis at a finer geographic resolution.^43^ All stores that reported data were included in our data set as they contained at least 90% of weekly data, a threshold used in previous work by Allaire et al.^43^ The final data set included 1,852 stores across 145 of the 254 counties in Texas.

### 2.3 BWN Occurrence Model

To evaluate the relationship between severe weather events and the issuance of BWNs, we implemented a Poisson regression model using balanced panel data across all Texas CWSs (*n* = 4673) and weeks, resulting in a CWS-week unit of analysis (eq 1). The model assumes the number of BWNs issued is a count variable following a Poisson distribution. The Poisson regression model was used for this analysis since BWN counts are discrete, non-negative integers, and the model effectively handles the skewed distribution typical of rare event counts, like severe weather data.^44^ The model includes fixed effects to control for time-invariant differences between CWSs and temporal trends.^45^ Standard errors were clustered at the CWS level to account for within-system correlation. The coefficients were exponentiated to produce incidence rate ratios (IRRs), which represent the multiplicative change in the expected rate of BWN issuance associated with a specific severe weather event.

1 $$\ln\left(\lambda_{ut}\right)=\sum_{j}B_{j}W_{utj}+\alpha_{uy}+\gamma_{t}$$

where: λ_ut_ is the expected number of BWNs issued for a specific CWS, *u*, for week, *t*; B_j_ is the estimated effect of weather type, *j*, on BWN count; *W*_utj_ is occurrence of severe weather type, *j*, in CWS, *u*, for week, t; α_uy_ is the fixed effect for CWS, *u*, in year, *y;* γ_t_ is the fixed effects for week, *t*.

### 2.4 BWN Duration Model

We modeled BWN duration using an unbalanced panel regression with fixed effects (eq 2), controlling for time-invariant differences between CWSs and temporal trends. CWSs that never issued a BWN during the time period were not included in the duration model as the duration would be 0 days for each entry. Furthermore, an entry without a valid rescind date was also excluded from duration analysis. An unbalanced panel data set was constructed to evaluate the effect of weather events on BWN duration, resulting in one CWS-week per BWN issued with a valid duration. The BWN duration was assigned to the utility-week in which the BWN was issued, and the associated duration was calculated based on the issue date and rescind date resulting in the entire BWN duration being accounted for in the week of issuance. Bootstrapped standard errors were computed using 1,000 replicates to ensure robustness and were clustered by CWS-year. Model selection was performed by rerunning the model with different variants of explanatory variables and fixed effects (SI Model Selection).

2 $$D_{ut}=\sum_{j}\beta_{j}W_{utj}+\alpha_{uy}+\gamma_{t}+\varepsilon$$

where: *D*_ut_ is the BWN duration (days) for a specific CWS, *u*, for week, *t*; β_j_ is the regression coefficient; *W*_utj_ is the occurrence of severe weather event type, *j*, in CWS, *u*, in week, *t;* α_uy_ is the fixed effect for a CWS, *u*, in year, *y*; γ_t_ is the fixed effect for week, *t;* and *ε* is the error term.

### 2.5 Bottled Water Sales Model

To evaluate community response to BWNs, we modeled the effect of BWNs on bottled water sales using a county-week, fixed-effects panel regression (eq 3), adapted from prior studies on health-based SDWA violations and consumer purchasing behavior.^43^ We included fixed effects for county-year (α_cy_) to absorb fixed differences between counties and year-to-year variation within counties, and week (γ_*t*_) fixed effects to absorb seasonal patterns common across counties. This analysis was conducted at the county level because it represents the finest spatial resolution available for the bottled water data.

3 $$\ln\left(S_{ct}\right)=\beta_{1}\left(\frac{k_{ict}}{7}\right)\left(\frac{p_{ic}}{p_{c}}\right)+\beta_{2}W_{ct}+\alpha_{cy}+\gamma_{t}+\varepsilon$$

where: *S*_ct_ is the bottled water sales for a given county, *c*, and week, *t;* β are regression coefficients; *k*_ict_ is the number of days the BWN violation was in effect in week *t*; p_ic_ is the population impacted by the BWN in county *c; p*_c_ is the population of county *c; W*_ct_ captures severe weather variables; α_cy_ is the fixed effects for a county, *c*, in a specific year, *y*; γ_t_ is the fixed effects for week *t*; and ε is the error term.

The BWN indicator ( $\left(\frac{k_{ict}}{7}\right)\left(\frac{p_{ic}}{p_{c}}\right)$ ) ranged from zero to one and was calculated as the product of (i) the fraction of the week the notice was active and (ii) the fraction of the county’s population served by affected CWSs. BWN indicators were summed when multiple BWNs occurred within the same county-week. For CWSs spanning multiple counties, service populations were proportionally distributed by shared area, assuming a homogeneous distribution. Since BWNs were aggregated to the county level for the bottled-water analysis, CWS specific variables were excluded from this model (i.e., size, owner, source). Severe weather variables were assigned to each county-week observation. Bootstrapped standard errors were calculated based on 1,000 repetitions with clusters by county-year.

### 2.6 Sensitivity Analyses

Sensitivity analyses were conducted using additional interaction terms in the duration model and bottled water sales model. As our models implement fixed effects to control for time-invariant characteristics (e.g., income, race), which are absorbed by the utility-time fixed effects, interaction terms and stratified subgroup analyses allowed us to observe how the effect of severe weather varies across different operational characteristics and sociodemographic groups. This approach accounts for confounding factors while simultaneously enabling evaluation of heterogeneous effects across characteristics. Interaction terms for the duration model were included for BWN cause, CWS operational characteristics (i.e., size, ownership, source) and sociodemographic characteristics. Specifically, interactions with rurality, income class, and racial distribution were used to examine the potential effect of community characteristics on the relationship between BWNs, severe weather events, and bottled water sales. The coefficients of the specific interactions were added to the reference level coefficients to obtain comparable estimates for each interaction.

### 2.7 Additional Statistical Analysis

All data processing, modeling, statistical analysis, and plotting was performed in *R* version 2025.05.1 + 513. Fixed-effects models used the “feols” function from the fixest package (version 0.8.4). To understand the impact of a large-scale, unprecedented weather event, the occurrence and duration models (Section 2.3 and 2.4, respectively) were also evaluated without BWNs issued during Winter Storm Uri. Wilcoxon pairwise tests were used to determine the statistical significance of BWN duration across weather events. Pearson correlations were used to assess associations between county population and both total and per capita sales.

## 3 Results and Discussion

### 3.1 1 BWN Issuance Patterns

Between 2010 to 2022, 66.6% of active CWSs in Texas (3,114 of 4,673) issued at least one BWN, with most issuances occurring in the most recent years (Figure 1A,C and Table 1). Approximately one out of ten CWSs across the state (8.5%, *n* = 398) issued more than 12 notices during the 13-year study period. Small CWSs accounted for the majority of all BWNs included in the occurrence model (69.0% of BWNs) and duration model (68.7% of BWNs) (Table 2). Publicly owned systems and groundwater-sourced systems were responsible for 65.7% and 63.9% of issued BWNs, respectively. The distribution of BWNs in Texas mirrored national studies of health-based SDWA violations, which also predominantly occurred in very small to small, groundwater-sourced systems.^33^ Most BWNs (∼85%) were attributed to distribution system issues such as low distribution pressure or water outages, suggesting infrastructure impacts rather than directly observed contamination (e.g., microbial) as the dominant driver. These findings are consistent with prior studies from Kentucky,^14,18^ West Virginia,^46^ and national data in 2021,^14^ where distribution problems were the leading cause of BWNs.

**Figure 1:**
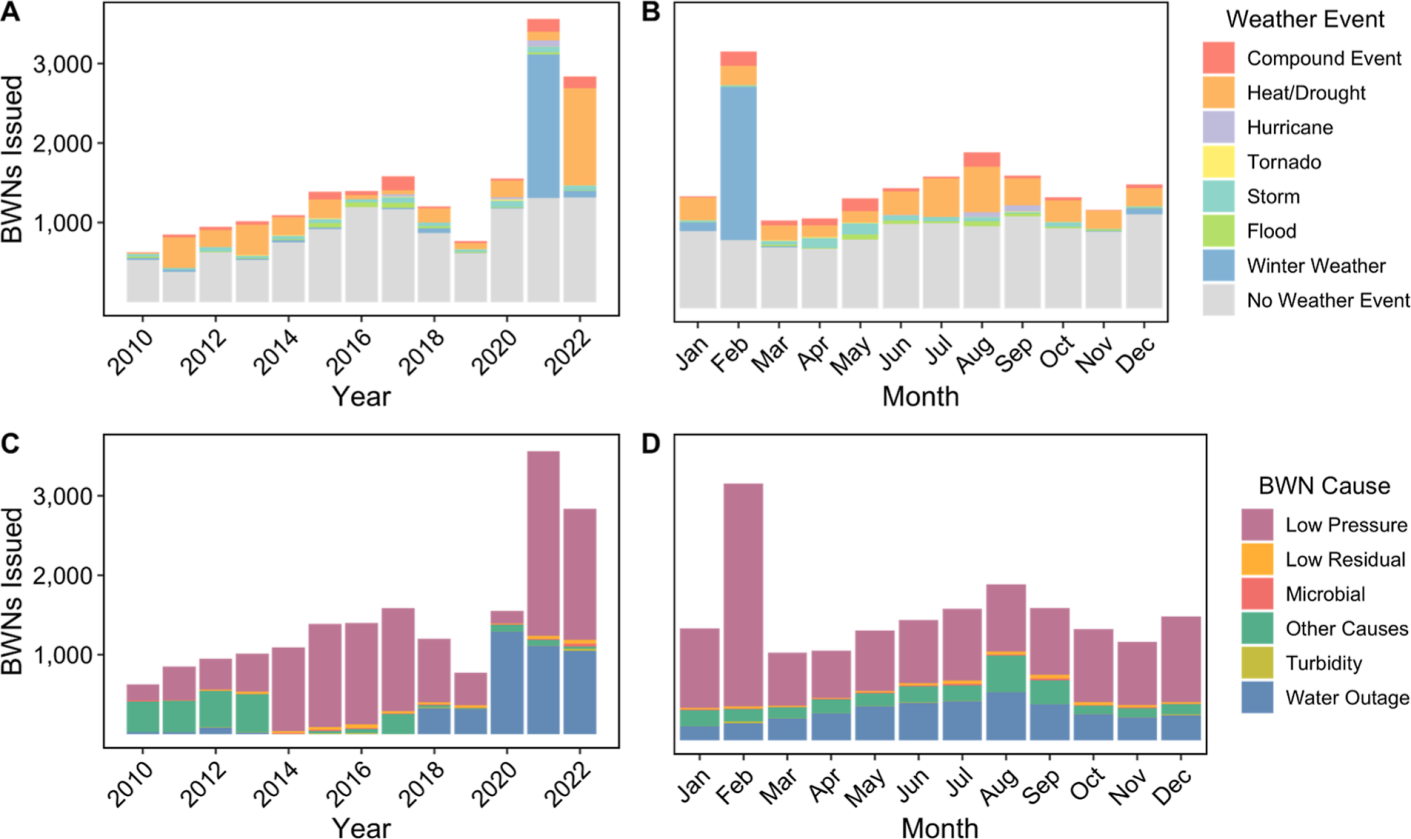
BWNs issued by coinciding weather events (A,B), and cause (C,D) for years and months, respectively. Figures include all BWNs issued in Texas (*n* = 18,839) which were included in the occurrence model.

**Table 2:** Summary of boil water notices included in the occurrence model and duration model

		occurrence model	duration model
		(*n* = 18,839)	(*n* = 18,276)
size	small	13,007 (69.0%)	12,549 (68.7%)
	medium/large	5,832 (31.0%)	5,727 (31.4%)
owner	private	6,459 (34.3%)	6,188 (33.9%)
	public	12,380 (65.7%)	12,088 (66.1%)
source	groundwater	12,039 (63.9%)	11,626 (63.6%)
	surface water	6,800 (36.1%)	6,650 (36.4%)
BWN cause	low distribution pressure	11,770 (62.5%)	11,429 (62.5%)
	low disinfection residual	339 (1.8%)	309 (1.7%)
	microbial	126 (0.7%)	117 (0.6%)
	turbidity	63 (0.3%)	61 (0.3%)
	water outage	4,261 (22.6%)	4,195 (23.0%)
	other	2,280 (12.1%)	2,165 (11.8%)
weather event	compound events	868 (4.6%)	788 (4.3%)
	heat & drought	3,356 (17.8%)	3,293 (18.0%)
	hurricane	143 (0.8%)	138 (0.8%)
	tornado	54 (0.3%)	54 (0.3%)
	storm	617 (3.3%)	600 (3.3%)
	flood	301 (1.6%)	272 (1.5%)
	winter weather	2,094 (11.1%)	2,086 (11.4%)
	no weather event	11,406 (60.5%)	11,045 (60.4%)

### 3.2 BWN Seasonality and Co-Occurrence with Severe Weather Events

BWN issuance in Texas exhibited clear seasonal trends, peaking in late summer (August) and showing secondary peaks in the winter (Figure 1B,D). Similar seasonal patterns have been reported in Kentucky,^18^ Canada,^47,48^ and Norway,^49^ where summer notices dominate. Increased BWN issuance in summer may reflect stressors such as hurricanes, heat, drought, and increased demand.^50^ BWNs associated with hurricanes primarily occurred in August and September (Figure 1B), coinciding with the peak months of the Atlantic hurricane season (June 1 to November 30).^51^

Overall, 39.5% of BWNs issued coincided with severe weather events, most commonly heat or drought (Table 2). The proportion of BWNs under these conditions was high during 2011–2014 and in 2022 (Figure 1A), coinciding with extended drought periods.^52^ Since relatively few BWNs were attributed to microbial or turbidity concerns (Table 2), the relationship between BWNs and heat or drought could reflect impacts to physical infrastructure rather than direct water quality drivers. Previous studies have linked drought to infrastructure issues including pressure loss, pump malfunction, and ineffective water treatment.^53–56^ Furthermore, drought periods are relatively long compared to other weather events (e.g., hurricanes) resulting in a higher potential for them to coincide with BWN issuances.

A notable event occurred during Winter Storm Uri in February 2021, when over 2,000 BWNs were issued in a single week which impacted ∼40% of CWSs statewide due to power outages, pipeline breaks, or other infrastructure failures (Figure 1A,B).^5,30,57^ BWNs issued during this time period were mostly due to low distribution pressure or water outage (84.7%, Table 2), which may or may not be related to power failures. The spike in BWN issuances during Winter Storm Uri underscores the vulnerability of water infrastructure to extreme winter weather events and also marked the start of a sharp rise in annual BWNs (Figure 1A). Although the reasons are unclear, the rise in notices may reflect increased awareness, improved reporting, or changes in operational practices after the storm. Additionally, it is possible the continued rise in BWNs reflects ongoing challenges resulting from Winter Storm Uri, including damages that have not yet been fully repaired. In a recent semistructured survey of large utilities in Texas a year after Winter Storm Uri, respondents highlighted concerns that small, resource-constrained systems may experience ongoing challenges resulting from Winter Storm Uri.^3^

We evaluated the co-occurrence of BWN issuance with severe weather by implementing a Poisson model. Weather events were associated with a greater occurrence of BWNs based on the results of our Poisson model with utility and time fixed effects (Figure 2). The analysis included all CWSs that were active in Texas throughout the time period, whether or not a BWN was issued. Winter weather, hurricanes, and floods resulted in a significant increase in the rate of BWN issuance while heat and drought, storms, and tornadoes had insignificant changes. Winter weather was associated with the highest percent rate increase (45%), followed by hurricanes (21%), and floods (3%). Notably, when BWNs issued during Winter Storm Uri were removed, the effect of winter weather was significantly reduced (20%, *p* < 0.05), indicating that the association with winter weather was largely driven by a single, albeit historical, weather event (Figure 2). This change highlights the significant scale of the impacts of Winter Storm Uri on water infrastructure. Heat and drought was the only weather event category that showed a decrease in the rate of BWN issuance (−0.03%), however this was not significant (95% confidence interval (CI): −1.3, 1.2%). The confidence intervals for hurricanes and winter weather were the largest of any weather event, likely due to their rarity relative to other events and varying intensity.

**Figure 2:**
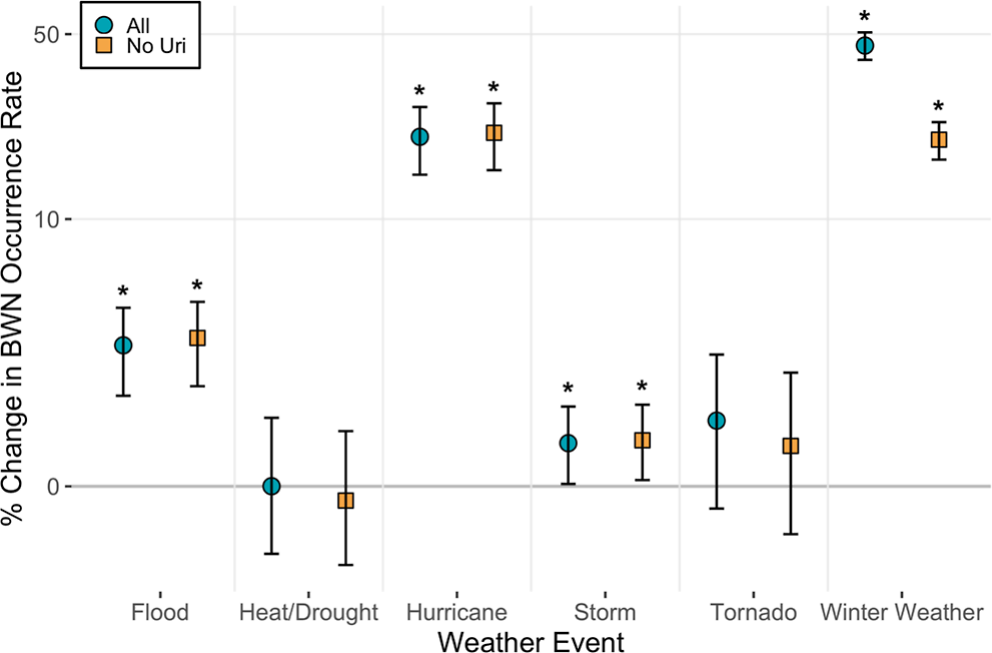
Percent change in the likelihood of BWN issuance from weather events. Points show the coefficient estimate, and error bars show the 95% confidence interval. *** represents a significance level *p* < 0.001.

These findings align with previous studies that have evaluated weather events and health-based SDWA violations. Previous studies have found that severe storms were associated with significant increases in SDWA violations,^58^ and extreme precipitation events resulted in increased detection of pathogens in drinking water systems.^59^ Overall, these findings suggest that future utility weatherization efforts may consider prioritizing winter weather, hurricanes, and floods to prevent issuance of BWNs.

### 3.3 BWN Duration and Duration Drivers

From 2010 to 2022, the mean BWN duration was 10.9 days, with most notices lasting 3–4 days (39.4%; Figure 3) showing the skewed distribution of BWN durations throughout the time span. Furthermore, 374 CWSs issued BWNs that lasted longer than 31 days, accounting for 3% of BWNs analyzed in the duration model, highlighting challenges in restoring access to safe water (Figure 3A,B). These prolonged BWNs disproportionately affected small CWSs (∼80%). Long-duration BWNs were concentrated in February of 2021, coinciding with Winter Storm Uri.

**Figure 3:**
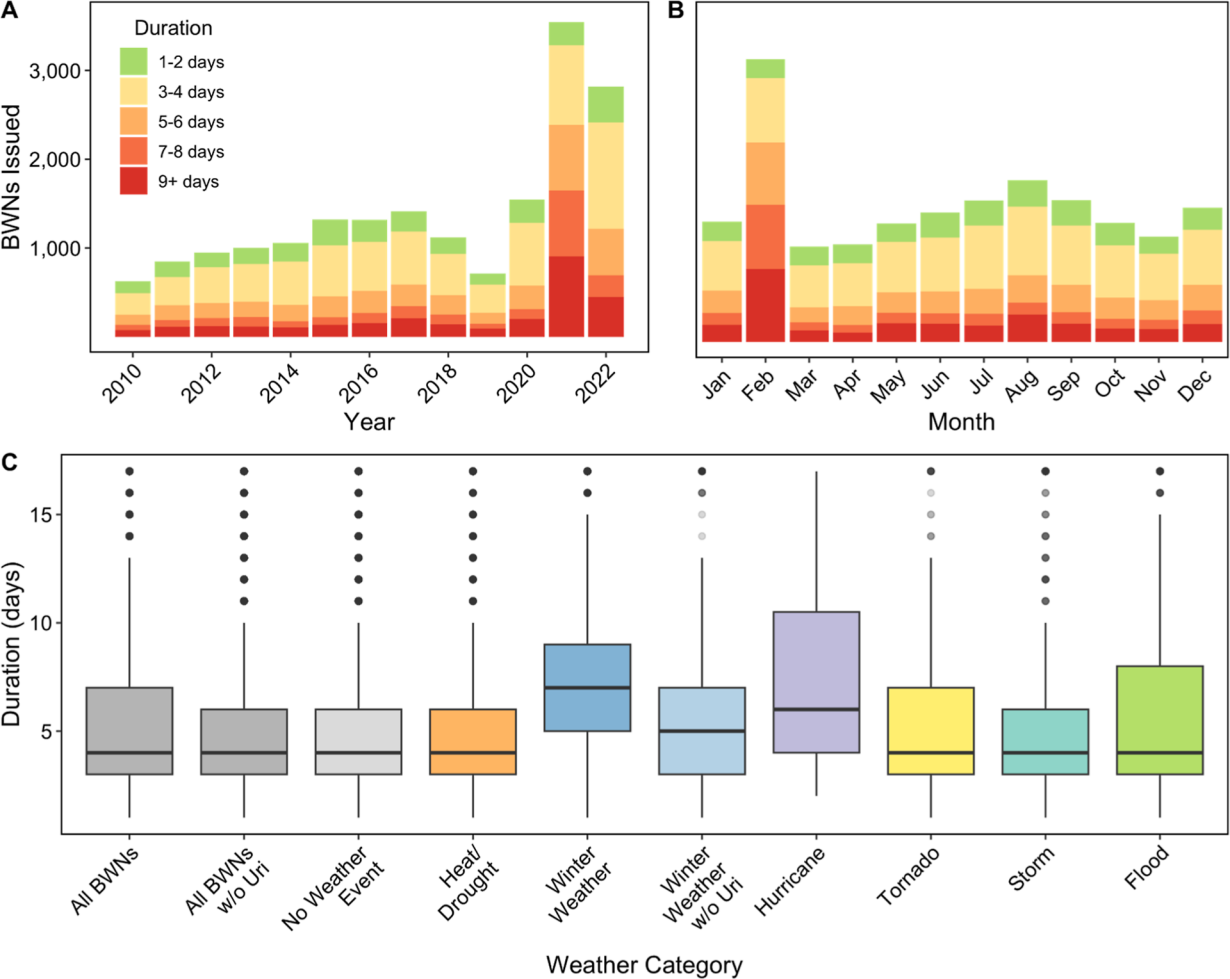
Duration of BWNs by year (A), month (B), and severe weather category (C). All BWNs and winter weather categories are shown with and without BWNs issued during Winter Storm Uri.

Winter weather was associated with the longest median BWN duration (7 days, *p* < 0.05) followed by hurricanes (6 days; Figure 3C). Floods, storms, tornadoes, and heat and drought had comparable median BWN durations compared to BWNs issued without a weather event. Excluding BWNs issued during Winter Storm Uri resulted in statistically significant reductions in median BWN duration during winter weather from 7 days to 5 days (−20%, *p* < 0.01) (Table S3). These findings underscore the disproportionate effect of a single extreme weather event in prolonging BWN duration and highlight resilience challenges faced by water infrastructure during unprecedented weather events like Winter Storm Uri.

We applied a fixed-effects regression model (eq 2, Section 2.4) on unbalanced panel data with controls for time invariant heterogeneity across CWSs to evaluate the environmental, operational, and sociodemographic predictors of BWN duration (Figures 4 and 5). Winter weather and hurricanes significantly increased BWN duration with increases of 1.6 (*p* < 0.001) and 1.1 (*p*< 0.05) days, respectively (Figure 4). Interestingly, these results suggest that while floods significantly increase the likelihood of a BWN being issued (Figure 2), they do not significantly impact the ability of a CWS to recover from a BWN. This highlights the importance of evaluating drinking water system resilience using multiple metrics, including both occurrence and duration of BWNs. When Winter Storm Uri was excluded from the model, winter weather no longer had a significant impact on BWN duration (Table S6). This again reflects the extent of Winter Storm Uri’s impact on CWSs in Texas. There was no significant impact on BWN duration due to heat and drought, tornadoes, floods, and storms. Since heat and drought typically occur for prolonged periods, it may be difficult to attribute BWNs to a specific heat or drought event which may impact estimated durations.

**Figure 4:**
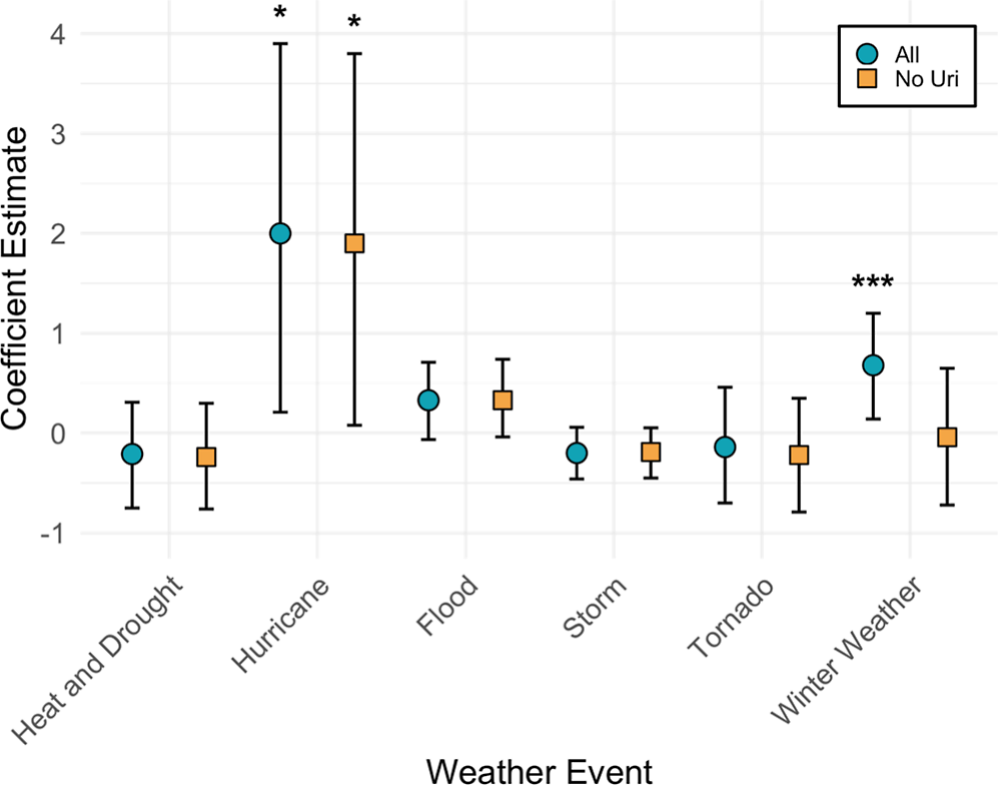
Coefficient estimates for the utility and time fixed effects model for the unbalanced panel set. Points show the coefficient estimate and the error bars show the 95% confidence interval based on bootstrapping (*n* = 1,000). Significance markers: *p* < 0.001: ***, *p* < 0.05: *.

**Figure 5:**
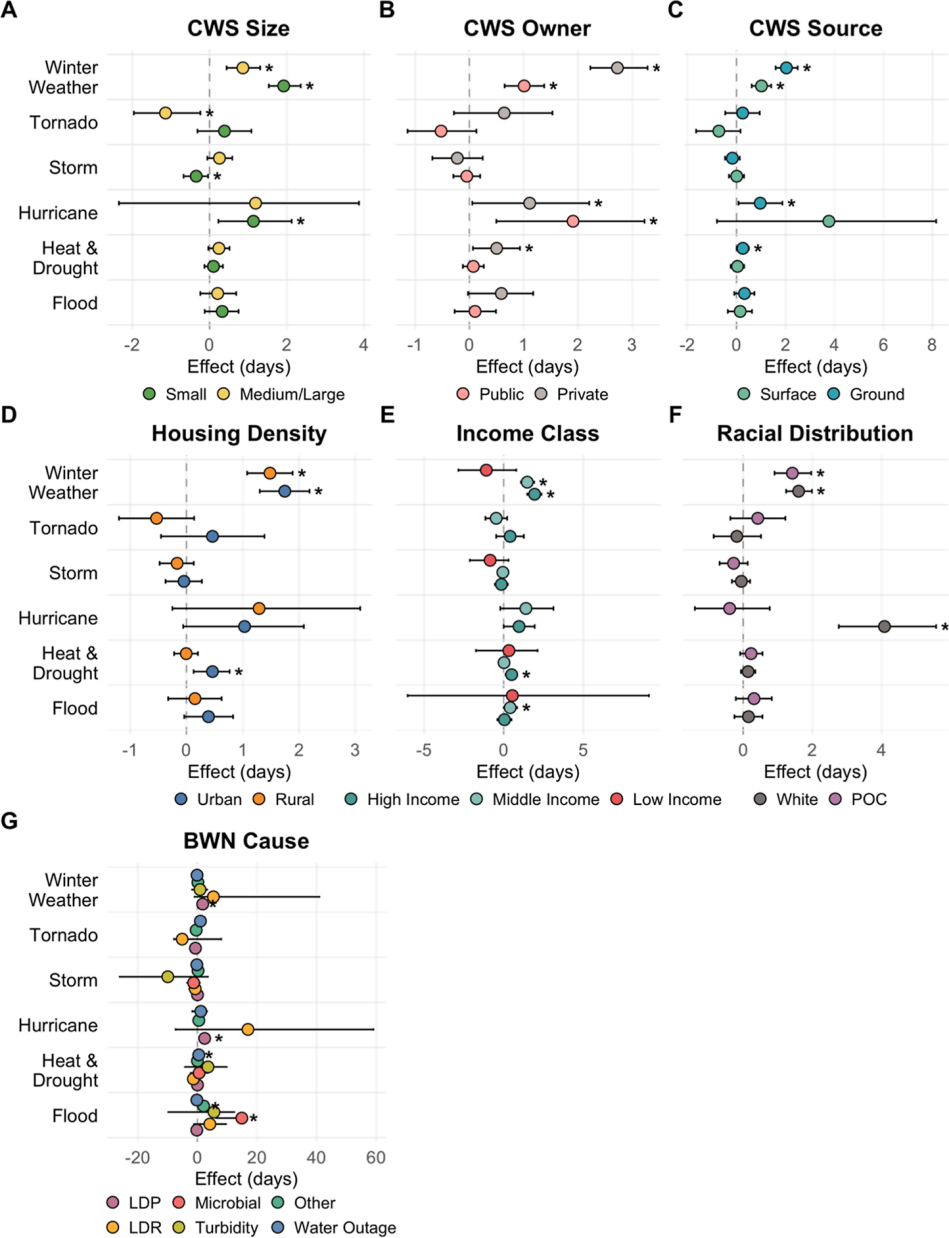
Interaction results for unbalanced panel data with Winter Storm Uri. Error bars show the 95% confidence intervals derived from bootstrapping (*n* = 1,000). Missing points are due to a lack of data in the original data set. Acronyms: POC: People of Color, LDP: Low Distribution Pressure, LDR: Low Disinfection Residual. * indicates significance (*p* < 0.05) based on bootstrapped confidence intervals. Estimates and confidence intervals are available in Tables S7–S13.

The reported cause of issuance was examined as an interaction term with weather events to assess how different causes contributed to the duration of BWNs (Figure 5, Table S7). Our findings revealed that certain causes were associated with significantly longer BWN durations under specific weather events. For example, for BWNs issued during floods, microbial contamination led to notices that were 9.3–15.1 days longer than all other operational causes (e.g., low distribution pressure). BWNs with a cause designated as ‘Other’ were significantly longer during floods (+2.1 days, 95% CI: 1.2, 3.0), indicating the cause did not fall within one of the five main categories. Similarly, low distribution pressure during hurricane BWNs (+2.4 days, 95% CI: 0.7, 4.1) and winter weather BWNs (+1.8 days, 95% CI: 1.4, 2.2) significantly increased the duration. BWNs attributed to low disinfection residual had the longest durations when they coincided with hurricanes, although the effect was not statistically significant (+16.9 days, 95% CI: −7.3, 59.0). Lastly, BWNs issued due to water outages significantly increased BWN duration during heat and droughts (+0.5 days, 95% CI: 0.2, 0.8). Since low distribution pressure and water outages can be related to power outages and there is no specific designation indicating power failure, future work should incorporate additional data to better assess its role. A key limitation of the BWN data is that causes such as power failures are not directly reported but are likely captured within the “low distribution pressure” and/or “water outage” categories, which together account for the majority of BWNs (84.7%, Table 2). Previous research has documented an increase in power-related failures during heat and drought, which may explain the observed significance with water outages.^60^ Similarly, analyses of power outages during Winter Storm Uri highlight the widespread and severe disruptions across Texas. Consistent with these findings, when BWNs issued during Winter Storm Uri are excluded from the model, low distribution pressure is no longer a significant predictor of BWN duration (Figure S3). These results suggest that power failures were an underlying driver of extended BWN durations, although additional analysis is needed to verify this relationship.

In summary, the underlying cause of a BWN interacts with the type of severe weather event to influence its duration, suggesting utilities may need cause-specific preparedness and response strategies to increase resilience to weather events. For example, low distribution pressure during hurricanes and winter weather likely reflects physical infrastructure failures such as pipe breaks, which require rapid infrastructure assessment and restoration of pressure before a notice can be lifted. In contrast, microbial contamination during floods necessitates resampling, treatment verification, and regulatory clearance, which cannot be accelerated through infrastructure repair alone. These distinctions indicate that resilience planning should move beyond generalized frameworks to incorporate response protocols tailored to the most likely failure modes associated with specific weather events. Targeted approaches could help reduce BWN duration and improve system resilience across different cause and weather event combinations.

### 3.4 Sociodemographic and Operational Predictors of BWN Duration

We implemented interaction terms on the duration model (eq 2, Section 2.6) for operational and sociodemographic characteristics to determine whether specific groups were more vulnerable to longer BWN durations during specific weather events (Figure 5, Tables S8–S13). Interactions revealed that no one operational or sociodemographic characteristic significantly influenced the duration of BWNs across all types of weather events. However, some operational and sociodemographic characteristics were associated with more pronounced effects on BWN duration under specific weather events.

Among operational parameters, small CWSs experienced significant increase in BWN duration for winter weather (+1.9 days, 95% CI: 1.5, 2.4) and hurricanes (+1.1 days, 95% CI: 0.23, 2.1), and significantly shorter BWNs for storms (−0.34 days, 95% CI: −0.67, −0.04) compared to BWNs that were not associated with weather events. For BWNs issued during winter weather events, the duration of the BWN increased more for small CWSs compared to BWNs issued by medium or large CWSs (+1.9 vs +0.86 days, *p* < 0.001). Small CWSs may have a more difficult time recovering from BWNs compared to large CWSs due to limited financial resources and workforce training.^61,62^ Previous studies also found that smaller CWSs experienced drinking water advisories with longer durations over long-term analysis in Kentucky,^18^ Canadian First Nation communities,^48^ and during Winter Storm Uri in Texas.^5^

Ownership type also produced significant interaction effects. Private CWSs experienced larger increases in BWN duration during winter weather (+2.7 days vs +1.0 days), heat and drought (+0.50 days vs +0.07 days), floods (+0.59 days vs +0.10 days) and tornadoes (+0.65 days vs −0.52 days) events compared to public CWSs, whereas public CWSs experienced the largest increase in BWN duration during hurricane events (+1.9 days vs +1.1 days). Groundwater-sourced CWSs exhibited the largest duration increase during winter weather (+2.0 days, 95% CI: 1.6, 2.5), hurricanes (+0.97 days, 95% CI: 0.08, 1.9), and heat and drought (+0.26 days, 95% CI: 0.01, 0.50), suggesting potential sensitivity to weather-driven fluctuations in source water quality or pumping capacity.^63,64^

Interactions for sociodemographic factors revealed that the effects of income class, housing density, and racial distribution on BWN duration estimates were similar across groups. CWSs in high-income counties had significant increases in BWN duration estimates during heat and drought events (+0.50 days, 95% CI: 0.12, 0.84), however these estimates were not significantly higher than those of CWSs in middle- and low-income counties. Similarly, CWSs in middle-income counties had significant increases in BWN duration estimates during floods (+0.40 days, 95% CI: 0.01, 0.83), but there was no significance between CWSs in high- and low-income counties. CWSs in high- and middle-income counties (+1.9 and +1.5 days, respectively) had significantly larger increases in BWN duration during winter weather events compared to CWSs in low-income counties (−1.1 days, *p* < 0.01), suggesting that the weather event modulates the relationship between income and duration.

CWSs in more urbanized counties experienced significantly larger BWN duration estimates during winter weather (+1.8 days vs +1.5 days), heat and drought (+0.5 days vs 0.0 days), and tornadoes (+0.5 days vs −0.5 days), showing that multiple weather types have a greater impact on CWSs in urban counties. Rural CWSs also showed a significant duration increase for winter weather (+1.5 days, *p* = 0.021). BWN durations for CWSs in both majority white and POC counties were significantly longer during winter weather +1.6 days (95% CI: 1.2, 2.0) and +1.4 days (95% CI: 0.9, 2.0), respectively, and during hurricanes in majority white counties (+4.1 days, 95% CI: 2.8, 5.6). These findings suggest that sociodemographic characteristics alone do not consistently predict BWN duration estimates.

### 3.5 Bottled Water Sales

Statewide, bottled water sales ranged from $2.3 million to $7.1 million per week and both total sales and per capita sales trended upward from 2010 to 2020 (Figure 6A). Sales typically peaked in summer months, with notable surges during the onset of the COVID-19 pandemic in March 2020. Severe weather events also correlated with sales increases, such as during Hurricane Harvey (August 2017) and Hurricane Laura (August 2020) (Figure 6A). Dallas County and Harris County, which have the highest housing densities, consistently reported the greatest per capita bottled water sales (Figure S4). Not surprisingly, county population was strongly correlated with overall sales (Pearson *R* = 0.93) and weakly correlated with per capita sales (Pearson *R* = 0.23), indicating that total bottled water sales are more closely tied to population size than individual behavior. The highest mean sales occurred in high-income (Figure 6B) and urban counties (Figure 6D), consistent with national patterns.^43,65,66^ We also found that majority white counties purchased more bottled water (Figure 6C). Conversely, previous studies found that Black and Hispanic residents are more likely to purchase bottled water than White residents due to increased distrust in public water quality.^67–69^

**Figure 6:**
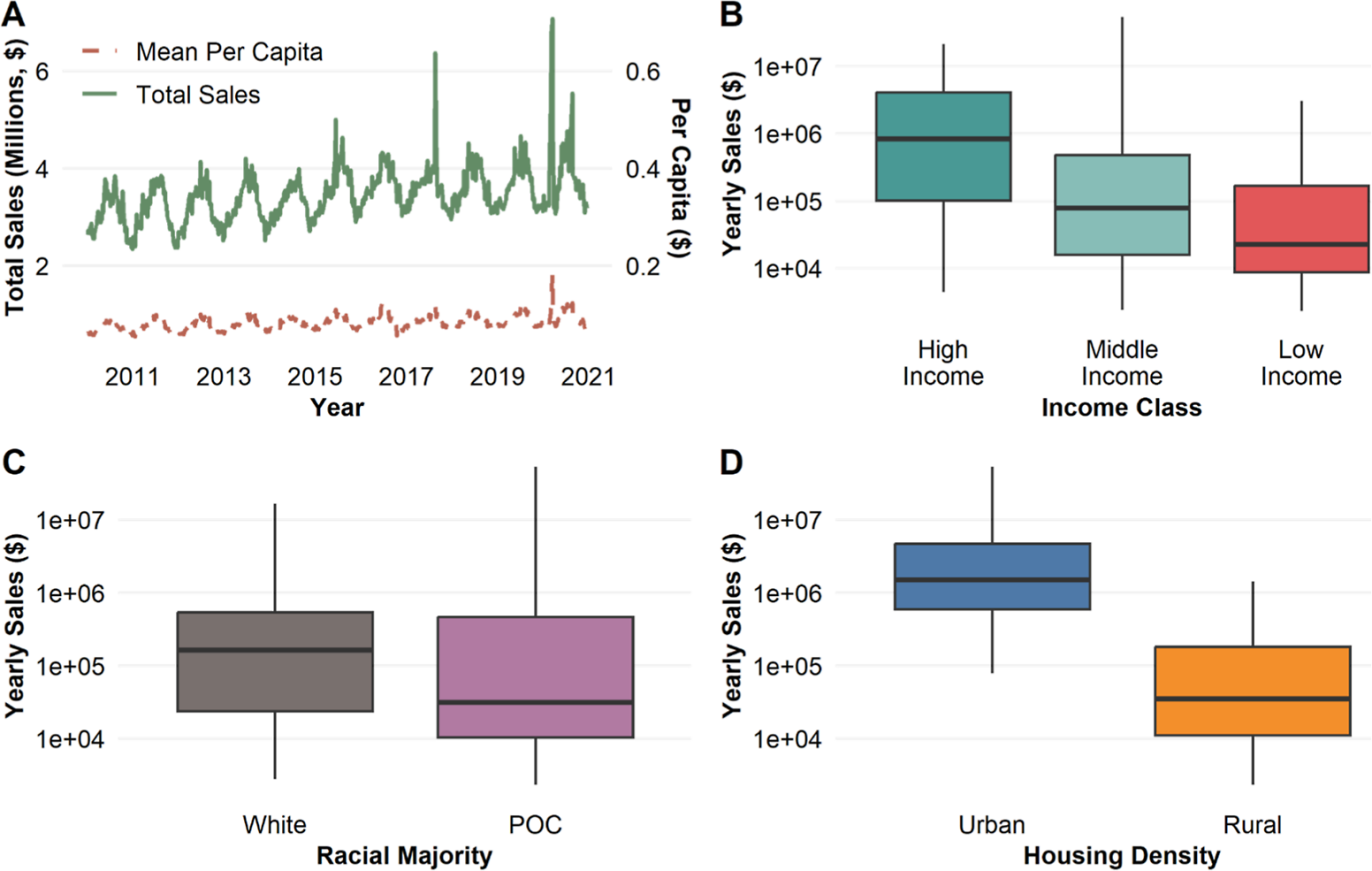
Bottled water sales in Texas aggregated at the county level including temporal trends in bottled water sales (A), and sales aggregated by income class (B), race (C), and housing density (D).

Community response is critical for minimizing health risks during BWNs. We found a significant 7.1% (95% CI: 1.9%–12.5%) increase in county-level bottled water purchases following BWN issuance (Table 3, eq 3, Section 2.5). Given that these notices often only affect subportions of a county, the localized pressure on retail supply chains in affected neighborhoods is likely substantially higher. Increasing bottled water sales suggest that households are taking actions to abide by BWNs by avoiding use of tap water. Our analysis only captured bottled water sales; however, households may be reliant on other averting behaviors (e.g., boiling water). Redundancy in averting strategies is key to improving resilience during severe weather events as boiling water may not be feasible during power or gas outages.

**Table 3:** Coefficient estimates on independent variables for bottled water sales and the percent change in aggregated sales^a^

independent variable	coefficient estimate	percent change (%)
BWN indicator	0.069** (0.019, 0.12)	7.1% (1.9, 12.5)
heat and drought	0.015*** (0.008, 0.022)	1.5% (0.8, 2.2)
winter weather	–0.026** (−0.037, −0.014)	–2.6% (−3.6, −1.4)
hurricane	0.16*** (0.10, 0.23)	17.4% (10.5, 25.9)
tornado	–0.0061 (−0.020, 0.0077)	–0.6% (−2.0, 0.8)
flood	–0.056*** (−0.065, −0.046)	–5.4% (−6.3, −4.5)
storm	–0.011** (−0.016, −0.0062)	–1.1% (−1.6, −0.6)

a Parentheses show the 95% confidence interval. Fixed effects for County-Year and Week were used. Significance levels are *** for 0.1% level, ** for 1% level, and * for 5% level.

Weather events also strongly influenced bottled water sales (Table 3). Bottled water sales increased (+5.9%) with a BWN and no weather event; however, there was a larger increase when a BWN was associated with a weather event (+11.6%) (Table S14). Out of all weather event types, hurricanes had the strongest increase in county-level bottled water sales (+17.7%) (Table 3), especially in higher-income counties (+24.1%, *p* < 0.05) (Figure 7). This could reflect preparedness for service disruptions or general preparedness for incoming weather rather than a response to BWNs themselves. Similarly, heat and drought led to a smaller, yet significant increase in bottled water sales (+1.6%). Conversely, floods were associated with lower bottled water sales, possibly due to sudden onset or access issues during flash flooding. Winter weather and storms were also associated with a significant reduction in bottled water sales indicating that households were less likely to purchase bottled water during these weather events.

**Figure 7:**
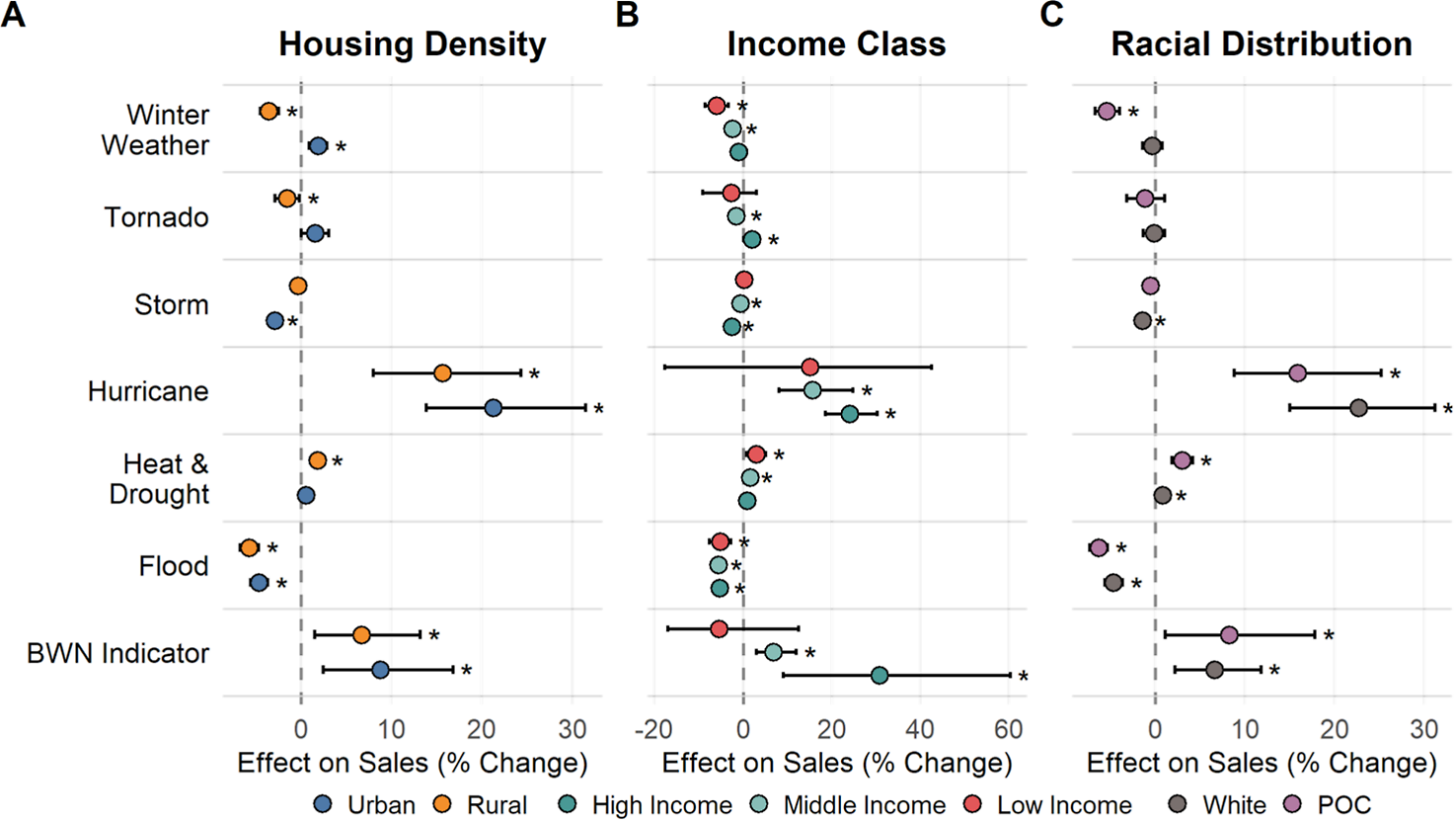
Effect of weather events and BWNs on bottled water sales. Points represent the estimates as the percent change in aggregated sales and the error bars show the 95% confidence interval. * indicates significant findings of *p* < 0.05. Estimates and confidence intervals are available in Tables S17–S19.

Sociodemographic interactions revealed important disparities in adaptive behavior during severe weather events and similar behaviors when a BWN is issued (Figure 7). Majority white counties increased bottled water purchases by 0.80% during heat and drought conditions, compared to a 2.9% increase in majority POC counties (*p* < 0.01). During winter weather, both majority white and majority POC counties reduced bottled water purchases; however, the reduction was approximately 5 percentage points greater in POC counties than in white counties (*p* < 0.001). During BWNs, majority POC counties had comparable increases in bottled water sales estimates compared to majority white counties (+8.2% and +6.6%, respectively). Furthermore, both majority white and majority POC counties significantly increased bottled water purchases during hurricanes (+22.6% for majority white counties and +15.9% for POC counties). These findings are interesting as previous research has shown that POC are generally more reliant on bottled water.66,68 The larger percentage increases observed in majority white counties may reflect differences in behavior during severe weather events. Nonsignificant impacts of BWN on bottled water sales in POC counties may indicate that the relative increase in bottled water sales during a severe weather event is lower due to a higher baseline bottled water purchasing. However, baseline bottled water sales in majority POC and white counties in this study were generally similar (Table S14), suggesting differences in model results are likely attributed to differences in county-level response to severe weather events rather than differences in baseline behavior.

Rural counties increased bottled water purchasing more than urban counties during heat and drought events (+1.8% and +0.52%, respectively). In contrast, rural counties decreased their bottled water purchasing (−3.4%) during winter weather while urban counties increased their bottled water purchasing (+1.9%) (*p* < 0.001) which may represent increased difficulties in access to stores during winter weather in rural areas relative to urban areas. Notably, high-income counties had the greatest increase in bottled water purchasing during a BWN (+30.9%), which was significantly higher than other income groups (*p* = 0.05), and low-income counties did not have a significant increase in bottled water sales (Figure 7). Furthermore, low-income counties did not have a significant increase in bottled water purchases during hurricanes where middle- and high-income counties did. It is possible that lower-income counties may face cost or access barriers that prevent them from purchasing bottled water or may opt for other compliance strategies such as boiling water. Previous studies have also shown price increases were heightened in lower-income communities during hurricanes when compared to high-income community pricing, signifying equity concerns.^70^ However, a previous survey-based study similarly found that higher-income groups were twice as likely to boil water compared to lower-income groups under BWNs during Winter Storm Uri.^30^ These findings underscore the need to better understand emergency water distribution strategies and risk communication in lower-income areas to support community resilience.

We show that bottled water data can be used to capture response to BWNs at large spatial scales. This represents a unique and useful feature of bottled water purchasing whereas other averting behaviors such as boiling water are difficult to track and quantify without household-level surveys. Here, we looked at robust data from one state to infer trends; however, our findings are transferable to regions facing similar weather risks. A potential limitation was that not all counties had data for bottled water sales, leading to them being excluded from our model. This study does not include information on donated water that may be distributed during severe weather events from public facilities such as shelters or churches, which may further limit holistic evaluation of averting measures taken by households. Previous survey-based studies showed there was a correlation between electric and gas utility availability and boiling water, especially when co-occurring disruptions shaped compliance to BWNs.^71^ While the model absorbs seasonal temperature patterns and heat and drought indirectly capture temperature, week-to-week temperature variations or anomalies were not explicitly included. Future work could integrate data from water, electricity, and gas utilities to offer a more comprehensive picture of cascading failures during weather events, assess the feasibility of different response strategies during emergencies, and characterize the overall resilience of utilities to inform where more efforts are needed.

### 3.6 Implications for Resilience

A better understanding of how severe weather events affect BWN duration and community response can guide both long-term infrastructure planning and short-term emergency response. This study presents the first regional-scale analysis of how severe weather affects BWN duration and community response, using bottled water sales as an indicator. Overall, the occurrence of BWNs disproportionately affects small, groundwater-sourced, publicly owned systems. The duration of BWNs is strongly influenced by the occurrence of severe weather events. Winter storms and hurricanes pose the greatest risk of prolonged BWNs, underscoring the need for climate resilience and weatherization of CWSs to these types of weather events.^3,5,72,73^ CWS size and ownership have a complex role in BWN duration, emphasizing that weatherization strategies must be tailored to account for specific CWS characteristics.

Current guidance for CWSs to combat severe weather events is reactive. For example, after Winter Storm Uri caused severe disruptions, CWSs in Texas were required to implement an Emergency Preparedness Plan which includes a backup power source, like a generator, in the event of power failure.^74^ Existing federal frameworks like the America’s Water Infrastructure Act 2018,^75^ which highlights resilience planning requirements, establish the precedent for planning but do not suggest how systems should prepare and respond to specific weather-driven disruptions. For example, system consolidation may appear to be a viable option for increasing climate resilience based on theincidence of BWNs alone. However, CWS size is often not a strong predictor of BWN duration, which represents the ability of a CWS to expediently address and rescind a BWN. The development of tiered, risk-based best practice frameworks that account for system size, source water type, and regional climate vulnerability could provide CWS operators and regulators a more actionable foundation than is currently available.

Once a BWN is issued, an increase in bottled water purchases was generally observed regardless of whether or not the BWN was associated with a weather event though this increase was more pronounced in higher-income groups. During hurricanes and in the absence of a BWN, higher-income, urban, and white communities were more likely to purchase bottled water. This suggests disparities in proactive emergency preparedness to severe weather but more similar behavior in response to BWNs. Efforts to improve equitable access to safe water supplies during a BWN can be improved by enhancing pre-disaster communication and preparedness. Disparities in emergency preparedness may be exacerbated if the anticipated weather event severely limits mobility or access to alternative averting strategies.

Lastly, combining BWN duration and bottled water sales provides a sociotechnical approach for evaluating drinking water system resilience that incorporates both physical infrastructure and community response. This approach can provide drinking water utilities, regulators, and public health agencies a tool to assess relative differences in community compliance to BWNs and accordingly prioritize emergency water supply plans. A key limitation is that this approach does not quantify the extent of the community complying with a BWN. Rather, it focuses on relative increases in bottled water purchases for a given county. However, understanding relative differences in response to BWNs and severe weather can help prioritize investments in CWS resilience, monitor changes in averting behavior during drinking water failures, and ensure equitable access to safe water during severe weather events and BWNs.

## Supplementary Material



## Notes

The Supporting Information contains additional model details of weather categories and variables, model selection, BWN duration breakdowns, model outputs including compiled data for incidence rate ratios and interactions, and figures excluding BWNs during Winter Storm Uri and county-level bottled water sales (PDF)

## References

[1] Ferdowsi A., Piadeh F., Behzadian K., Mousavi S.-F., Ehteram M. (2024). Urban Water
Infrastructure: A Critical Review on Climate Change Impacts and Adaptation
Strategies. Urban Climate.

[2] Inwald J. F., Bruine De Bruin W., Yaggi M. (2025). Disaster Preparedness and Perceptions
of People Who Experienced Water Insecurity: Insights from the Lloyd’s
Register Foundation’s World Risk Poll. Environ. Sci. Technol..

[3] Tiedmann H. R., Spearing L. A., Castellanos S., Stephens K. K., Sela L., Faust K. M. (2023). Tracking the Post-Disaster Evolution of Water Infrastructure
Resilience: A Study of the 2021 Texas Winter Storm. Sustainable Cities and Society.

[4] TCEQ . TCEQ Plan: After-Action Review of Public Water Systems and Winter Storm Uri. https://www.tceq.texas.gov/downloads/publications/gi/gi-598.pdf (accessed 2025–09–16).

[5] Glazer Y. R., Tremaine D. M., Banner J. L., Cook M., Mace R. E., Nielsen-Gammon J., Grubert E., Kramer K., Stoner A. M. K., Wyatt B. M., Mayer A., Beach T., Correll R., Webber M. E. (2021). Winter Storm Uri: A Test of Texas’ Water Infrastructure
and Water Resource Resilience to Extreme Winter Weather Events. J. of Extr. Even..

[6] Safarpour H., Spearing L. A. (2024). Temporal Public Perceptions and Experiences during
Water Service Disruptions: The Case of Jackson, Mississippi. Environ. Res.: Infrastruct. Sustain..

[7] America’s Water Infrastructure Act of 2018, Pub. L. No. 115-270, 132 Stat. 3881 (2018). U.S. Government Publishing Office. https://www.govinfo.gov/content/pkg/COMPS-15473/pdf/COMPS-15473.pdf (accessed July 11, 2024).

[8] Committee on Increasing National Resilience to Hazards and Disasters; Committee on Science, Engineering, and Public Policy; Policy and Global Affairs; National Academies. Disaster Resilience: A National Imperative; National Academies Press: Washington, DC, 2012. https://www.nationalacademies.org/read/13457 (accessed July 11, 2024).

[9] LaPatin M., Ritsch N., Armanios D. E., Albertson L., Katz L. E., Faust K. M. (2024). A Stakeholder-Systems
Analysis of
Water Provision in Rural Alaska. ACS EST Water.

[10] Balaei B., Wilkinson S., Potangaroa R., McFarlane P. (2020). Investigating
the Technical Dimension of Water Supply Resilience to Disasters. Sustainable Cities and Society.

[11] Thelemaque N., Spearing L. A., Faust K. M., Kaminsky J. A. (2023). Small Drinking Water
Utilities’ Resilience: The Case of the COVID-19 Pandemic. ACS EST Water.

[12] Toland, J. ; Spearing, L. A. Water Utilities and Equity in Disasters: A Systematic Literature Review. In ASCE Inspire 2023; American Society of Civil Engineers: Arlington, Virginia, 2023; pp 349–357. 10.1061/9780784485163.042.

[13] Opdyke A., Javernick-Will A., Koschmann M. (2017). Infrastructure
Hazard Resilience
Trends: An Analysis of 25 Years of Research. Nat. Hazards.

[14] U.S Environmental Protection Agency . National Occurrence and Causes of Boil Water Advisories in the United States Report to Congress, 2024. EPA 810-R-24–003. https://www.epa.gov/system/files/documents/2025-01/10586_boil-water-advisories_final_rtc_20240603_admin.pdf.

[15] Texas Commission on Environmental Quality. 30 Tex. Admin . Code § 290.122: Public Drinking Water; Texas Administrative Code, Title 30, Part 1, Chapter 290. https://texas-sos.appianportalsgov.com/rules-and-meetings?chapter=290&interface=VIEW_TAC&part=1&title=30 (accessed July 11, 2024).

[16] O’Shay S., Day A. M., Islam K., McElmurry S. P., Seeger M. W. (2022). Boil Water Advisories
as Risk Communication: Consistency
between CDC Guidelines and Local News Media Articles. Health Communication.

[17] Reynolds, K. Boil Water Notices in the U.S. 2012–2014; Water Quality Research Foundation; Zuckerman College of Public Health, University of Arizona: Tucson, AZ, 2016. https://www.vettersculliganwater.com/content/documents/vetters/boil-water-alerts.pdf (accessed August 10, 2025).

[18] Alzahrani F., Tawfik R. (2024). Public Water Service Disruptions: A Descriptive Analysis
of Boil Water Advisories. Water.

[19] Lyle Z. J., VanBriesen J. M., Samaras C. (2023). Drinking Water Utility-Level Understanding
of Climate Change Effects to System Reliability. ACS EST Water.

[20] Bondank E. N., Chester M. V., Ruddell B. L. (2018). Water Distribution System Failure
Risks with Increasing Temperatures. Environ.
Sci. Technol..

[21] Mulki S., Rubinstein C., Saletta J. (2018). Texas’ Water Quality Challenge
and the Need for Better Communication in an Era of Increasing Water
Quality Contamination Events. Tex. Wat. J..

[22] Mullin M. (2020). The Effects
of Drinking Water Service Fragmentation on Drought-Related Water Security. Science.

[23] Laucelli D., Giustolisi O. (2015). Vulnerability Assessment of Water Distribution Networks
under Seismic Actions. J. Water Resour. Plann.
Manage..

[24] Wright B., Stanford B. D., Reinert A., Routt J. C., Khan S. J., Debroux J. F. (2014). Managing Water Quality
Impacts from Drought on Drinking
Water Supplies. J. Water Supply:Res. Technol.--AQUA.

[25] Benotti M. J., Stanford B. D., Snyder S. A. (2010). Impact
of Drought on Wastewater Contaminants
in an Urban Water Supply. J. of Env Quality.

[26] Mosley L. M. (2015). Drought
Impacts on the Water Quality of Freshwater Systems; Review and Integration. Earth-Sci. Rev..

[27] Grineski S. E., Collins T. W., Chakraborty J. (2022). Cascading
Disasters and Mental Health
Inequities: Winter Storm Uri, COVID-19 and Post-Traumatic Stress in
Texas. Social Science & Medicine.

[28] Islam K., Laskey A., Schwetschenau S., Sheppard R., Smith R., Sobeck J., Stanley E., Taylor K., Kilgore P., Love N., McElmurry S., Seeger M. (2023). Crisis Management of
Interdependent Systems, Communication and Coordination: A Perspective
on Medium-scale Events. Contingencies &
Crisis Mgmt.

[29] Vedachalam S., Spotte-Smith K. T., Riha S. J. (2016). A Meta-Analysis of Public Compliance
to Boil Water Advisories. Water Res..

[30] Day A. M., O’Shay S., Islam K., Seeger M. W., Sperone F. G., McElmurry S. P. (2024). Boil Water
Notices as Health-Risk Communication: Risk
Perceptions, Efficacy, and Compliance during Winter Storm Uri. Sci. Rep.

[31] Rundblad G., Knapton O., Hunter P. R. C. (2010). Perception
and Behaviour during a
Natural Disaster Involving a “Do Not Drink” and a Subsequent
“Boil Water” Notice: A Postal Questionnaire Study. BMC Public Health.

[32] Texas Commission on Environmental Quality . State of Texas Public Drinking Water Program: 2022 Annual Compliance Report; Office of Water, Water Supply Division; Texas Commission on Environmental Quality: Austin, TX, 2023. https://www.tceq.texas.gov/downloads/drinking-water/epa-acr-2022.pdf (accessed July 11, 2024).

[33] U.S. Environmental Protection Agency . Compliance Evaluation of Community Water Systems in the United States Using Data from the Safe Drinking Water Information System; EPA 815-R-24-031; U.S. Environmental Protection Agency: Washington, DC, 2024. https://www.epa.gov/system/files/documents/2025-01/final_508_epa_reporttocongress_11-19-24_0.pdf (accessed July 11, 2024).

[34] National Oceanic and Atmospheric Administration, National Centers for Environmental Information . Storm Events Database: Storm Event Details, 2010–2022; https://www.ncei.noaa.gov/pub/data/swdi/stormevents/csvfiles/ (accessed July 11, 2024).

[35] Texas Water Development Board . Water Service Boundary Viewer Shapefile Dataset; https://www3.twdb.texas.gov/apps/waterserviceboundaries (accessed 2024–07–11).

[36] U.S. Environmental Protection Agency . Safe Drinking Water Information System (SDWIS) Dataset; https://www.epa.gov/enviro/sdwis-overview (accessed July 1, 2024).

[37] U.S. Census Bureau . Small Area Income and Poverty Estimates (SAIPE) Program Dataset, 2010–2022;https://www.census.gov/programs-surveys/saipe/data/datasets.html (accessed July 1, 2024).

[38] Community Reinvestment Act of 1977, Pub. L. No. 95-128, §§ 801-804, 91 Stat. 1147 (codified as amended at 12 U.S.C. §§ 2901-2906).

[39] U.S. Census Bureau . Population and Housing Unit Estimates Dataset. https://www.census.gov/programs-surveys/popest/data/data-sets.html (accessed 2024–07–01).

[40] Texas Open Data Portal; U.S. Census Bureau. Texas Counties Cartographic Boundary Map. https://data.texas.gov/dataset/Texas-Counties-Cartographic-Boundary-Map/sw7f-2kkd/about_data (accessed 2024–07–01).

[41] Housing Assistance Council . Hac’s Rural & Small Town Typology Database. Version: HAC Tract-9 January 2014. https://ruralhome.org/wp-content/uploads/storage/documents/policy_comments/dts/TECHNICAL_DOCUMENTATION_HAC_Rural__Small_Town_Definition.pdf. (accessed 2025–08–23).

[42] U.S. Census Bureau , Understanding and Using American Community Survey Data: What All Data Users Need to Know, U.S. Government Publishing Office, Washington, DC, 2020.

[43] Allaire M., Mackay T., Zheng S., Lall U. (2019). Detecting Community
Response to Water Quality Violations Using Bottled Water Sales. Proc. Natl. Acad. Sci. U.S.A..

[44] McDonnell K. A., Holbrook N. J. (2004). A Poisson Regression Model of Tropical
Cyclogenesis
for the Australian–Southwest Pacific Ocean Region. Wea. Forecasting.

[45] Imai K., Kim I. S. (2019). When Should We Use
Unit Fixed Effects Regression Models
for Causal Inference with Longitudinal Data?. American J. Political Sci..

[46] Alzahrani F., Collins A. R. (2022). Impact of Public Water Supply Unreliability on Residential
Property Prices in Marion County, West Virginia. Agric. Resour. Econom. Rev..

[47] Moghaddam-Ghadimi S., Tam A., Khan U. T., Gora S. L. (2023). How Might Climate Change Impact Water
Safety and Boil Water Advisories in Canada?. FACETS.

[48] Thompson E. E., Post Y. L., McBean E. A. (2017). A Decade
of Drinking Water Advisories:
Historical Evidence of Frequency, Duration and Causes. Canadian Water Resources Journal/Revue canadienne des ressources
hydriques.

[49] Hyllestad S., Veneti L., Bugge A. B., Rosenberg T. G., Nygård K., Aavitsland P. (2019). Compliance
with Water Advisories
after Water Outages in Norway. BMC Public Health.

[50] Rahman G., Jung M.-K., Kim T.-W., Kwon H.-H. (2025). Drought Impact,
Vulnerability, Risk Assessment, Management and Mitigation under Climate
Change: A Comprehensive Review. KSCE Journal
of Civil Engineering.

[51] Islam T., Merrell W., Seitz W., Harriss R. (2009). Origin, Distribution,
and Timing of Texas Hurricanes: 1851–2006. Nat. Hazards Rev..

[52] Ferencz S. B., Sun N., Turner S. W. D., Smith B. A., Rice J. S. (2024). Multisectoral Analysis
of Drought Impacts and Management Responses to the 2008–2015
Record Drought in the Colorado Basin, Texas. Nat. Hazards Earth Syst. Sci..

[53] Goodin, A. Drought Response, Recovery and Resilience: A Guide for Public Water Systems - PUB2745, 2019. https://dnr.mo.gov/document-search/drought-response-recovery-resilience-guide-public-water-systems-pub2745/pub2745. (accessed 2025–10–13).

[54] NIDIS . National Integrated Drought Information System: Water Utilities, 2025. https://www.drought.gov/sectors/water-utilities.

[55] Mullin M. (2020). The Effects
of Drinking Water Service Fragmentation on Drought-Related Water Security. Science.

[56] Howard G., Beevers L., Charles K., Nijhawan A. (2026). The Vulnerability and
Resilience of Drinking Water Systems to Extreme Weather Events and
Future Climate Change. Curr. Envir Health Rpt.

[57] Melaku N. D., Fares A., Awal R. (2023). Exploring
the Impact of Winter Storm
Uri on Power Outage, Air Quality, and Water Systems in Texas, USA. Sustainability.

[58] Chen X., Fisk J. M., Mayer M. K., McNamara M. W., Morris J. C. (2024). Two’s
a Company, Three’s a Cloud: Explaining the Effect of Natural
Disasters on Health-based Violations in Drinking Water. Risk Hazard & Crisis Pub Pol.

[59] Austin, W. ; Pan, S. ; Parthum, B. M. When It Rains, It Pours: Severe Weather Events, Flooding, and Drinking Water Quality. NCEE Working Paper 24-12 2024. https://www.epa.gov/system/files/documents/2024-12/2024-12.pdf

[60] Saki S., Sofia G., Kar B., Anagnostou E. (2025). A Multi-Year
Analysis of the Impact of Heatwaves and Compound Weather Events on
Power Outages. Sci. Rep.

[61] Milman A., Ashjian James O., Macuch C. (2023). Motivating the Formation
of Partnerships
by Small Water Systems. Utilities Policy.

[62] McFarlane K., Harris L. M. (2018). Small Systems, Big Challenges: Review
of Small Drinking
Water System Governance. Environ. Rev..

[63] Nielsen-Gammon J. W., Banner J. L., Cook B. I., Tremaine D. M., Wong C. I., Mace R. E., Gao H., Yang Z., Gonzalez M. F., Hoffpauir R., Gooch T., Kloesel K. (2020). Unprecedented Drought
Challenges for Texas Water Resources in a Changing Climate: What Do
Researchers and Stakeholders Need to Know?. Earth’s Future.

[64] Asif Z., Chen Z., Sadiq R., Zhu Y. (2023). Climate Change Impacts
on Water Resources and Sustainable Water Management Strategies in
North America. Water Resour Manage.

[65] Cohen A., Zhang Q., Luo Q., Tao Y., Colford J. M., Ray I. (2017). Predictors of Drinking Water Boiling
and Bottled Water Consumption
in Rural China: A Hierarchical Modeling Approach. Environ. Sci. Technol..

[66] Hu Z., Morton L. W., Mahler R. L. (2011). Bottled Water: United States Consumers
and Their Perceptions of Water Quality. IJERPH.

[67] Colburn A. T., Kavouras S. A. (2021). Tap Water Consumption
and Perceptions in United States
Latinx Adults. Nutrients.

[68] Jaffee D. (2024). Unequal Trust:
Bottled Water Consumption, Distrust in Tap Water, and Economic and
Racial Inequality in the United States. WIREs
Water.

[69] Javidi A., Pierce G. U. S. (2018). Households’
Perception of Drinking Water as
Unsafe and Its Consequences: Examining Alternative Choices to the
Tap. Water Resour. Res..

[70] Barriola X., Schmidt W. (2025). The High Impact of Hurricanes on
Prices in Low-Income
Communities. M&SOM.

[71] Day A. M., Islam K., O’Shay S., Taylor K., McElmurry S. P., Seeger M. W. (2022). Consumer Response
to Boil Water Notifications During
Winter Storm Uri. J. AWWA.

[72] Matthews J. C. (2016). Disaster
Resilience of Critical Water Infrastructure Systems. J. Struct. Eng..

[73] Becher O., Smilovic M., Verschuur J., Pant R., Tramberend S., Hall J. (2024). The Challenge of Closing the Climate Adaptation Gap for Water Supply
Utilities. Commun. Earth Environ.

[74] Texas Water Code § 13.1394. Standards of Emergency Operations (2025). https://statutes.capitol.texas.gov/?tab=1&code=WA&chapter=WA.13&artSec=13.1394. (accessed October 15, 2025).

[75] Klobuchar, A. America’s Water Infrastructure Act of 2018, Pub. L. No. 115-270, 132 Stat. 3765 (2018). https://www.govinfo.gov/content/pkg/COMPS-15473/pdf/COMPS-15473.pdf (accessed July 11, 2024).

