# Supplementary material for: Evaluating the Resilience of Drinking Water Systems to Severe Weather Events Using Boil Water Notices and Bottled Water Sales
Source: Environ Sci Technol. 2026 Jul 31;60(31):21648–60. doi: 10.1021/acs.est.5c18579 (PMC13472146; doi:10.1021/acs.est.5c18579)
Supplement: Supplementary file 1 [file es5c18579_si_001.pdf]

Supporting Information: Evaluating the Resilience of Drinking Water Systems to Severe Weather Events Using Boil Water Notices and Bottled Water Sales

Marlee Shaffer[a], Nora Awad[a], Frances Davenport[b], and Sarah Fakhreddine[a*]

[a]Department of Civil and Environmental Engineering, Carnegie Mellon University, PA 15213, USA

[b]Department of Civil and Environmental Engineering, Colorado State University, Fort Collins, CO, 80523, USA

*Corresponding Author: Sarah Fakhreddine

19 pages, 4 Figures, and 19 Tables

Table S1: Weather Event Groupings
Table S2: Model Variables Descriptions and Data Source
Figure S1: Sensitivity Analysis for Connecting CWS to County or NWS Zone
Figure S2: Duration Model BWN Causes and Co-occurring Weather.
Table S3: Mean Durations of BWNs for Various Weather Events
Table S4: IRR and Percent Change for Various Fixed-Effects for the Poisson Model.
Table S5: OLS Duration Model with All Sociodemographic and Operational Variables
Table S6: BWN Duration Model Comparison with Varying Fixed Effects on Unbalanced Data
Table S7: Duration Interaction Results for BWN Cause
Table S8: Duration Interaction Results for CWS Size
Table S9: Duration Interaction Results for CWS Owner
Table S10: Duration Interaction Results for CWS Source
Table S11: Duration Interaction Results for Housing Density
Table S12: Duration Interaction Results for Income Class
Table S13: Duration Interaction Results for Racial Distribution
Figure S3: Duration Model Interaction Results Excluding Winter Storm Uri
Figure S4: County Level Total Sales and Per Capita Sales
Table S14: Model Results for Bottled Water for Weather Events and BWNs
Table S15: Mean Baseline Bottled Water Sales in Majority POC vs. Majority White Counties
Table S16: Model Results for Bottled Water Model.
Table S17: Sales Interaction Results for Housing Density
Table S18: Sales Interaction Results for Income Class
Table S19: Sales Interactions Results for Racial Distribution

Table S1. Weather Event Groupings

Weather Grouping	Specific Weather Events
**Heat and Drought**	Heat, Drought, Wildfire
**Winter Weather**	Snow, Cold, Sleet, Ice, Winter, Freeze
**Hurricane**	Hurricane, Tropical, Surge
**Tornado**	Tornado, Funnel
**Storm**	Hail, Rain, Thunderstorm, Lightning, Strong Winds, High Winds
**Flood**	Flood, Flash Flood, Coastal Flood

Table S2. Model Variables Description and Data Source

Variable	Description	Categories / Units	Notes
**BWN Duration (D_{ut})**	Number of days between issuance and rescind date of a boil water notice	Units: Days	Calculated from the start and end date in the TCEQ dataset
**Cause of BWN**	Primary reason for BWN issuance	- Low distribution pressure<br>- Low disinfectant residual<br>- Microbial contamination<br>- Turbidity<br>- Water outage<br>- Other	As reported in TCEQ dataset
**Severe Weather (W_{ct})**	Indicator for extreme weather events	Binary: 1 = weather event present, 0 = none	Based on NOAA database Weather types grouped (see Table S1)
**CWS Size**	Utility size based on population served	- Small (<500–3,300)<br>- Large (3,300 >10,000)	Based on EPA SDWIS categories
**Income Class**	Community income	- Low- and moderate income (<80% AMI)<br>- Middle-income (80% to 120% AMI)<br>- High-income (>120% AMI)	From Census SAIPE; inflation-adjusted
**Racial Composition**	Percent of population by race	- % Black<br>- % Hispanic<br>- % Asian<br>- % Other	"Other" includes multiracial and Native American populations
**Housing Density**	Number of housing units per square mile	Units: units/mi²<br>- Urban (>64 units/mi²)<br>- Rural (<64 units/mi²)	Derived from Census housing and land area data
**BWN Indicator ($k_{itc}p_{ic}/7p_c$)**	Weighted BWN in a given county and week	Value from 0 to 1	Fraction of county affected × fraction of week affected
**Bottled Water Sales (S_{ct})**	Weekly bottled water sales	ln-transformed $ sales	From NielsenIQ Retail Scanner dataset

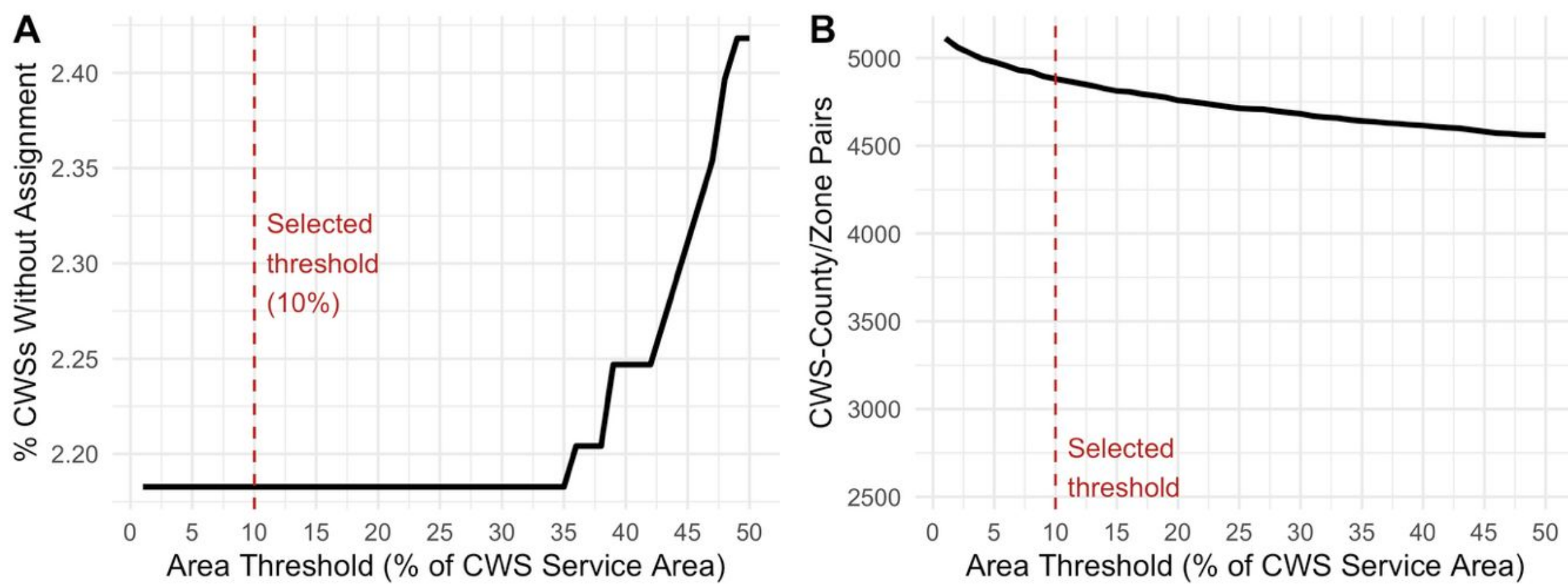

Figure S1. Sensitivity Analysis for Connecting CWS to County or NWS Zone. A) Percent of CWSs that are not assigned a county or zone at each tested area threshold. B) CWS-County or Zone Pairs for each area threshold.

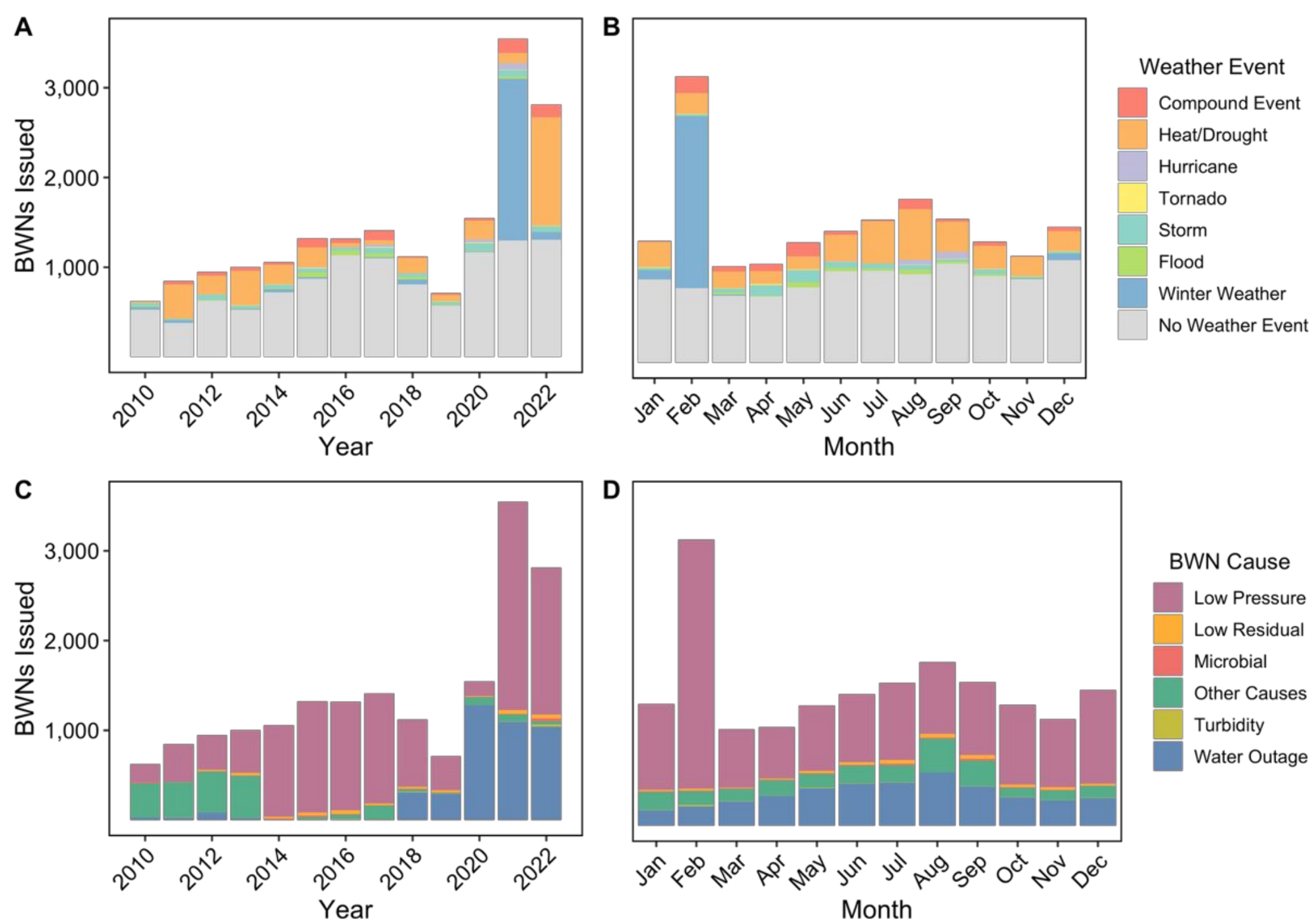

Figure S2. Breakdowns of A&B) Weather events coinciding with BWNs and C&D) BWN cause based on BWNs used in the Duration Model (Section 2.4)

Table S3. Mean Durations of BWNs for Various Weather Events. This data is based on the calculated duration from BWN issuance and was calculated using the raw data and the duration data that has been winsorized. Comparing raw duration to winsorized data shows the length disparity between certain events caused by only a few BWNs as the median durations were the same. The – indicates there was no change between the datasets. See Section 2.4 for details.

BWN Category	Raw Duration (mean ± se) All BWNs	Raw Duration (mean +/- se) Winter Storm Uri Removed	Winsorized Duration (mean ± se) All BWNs	Winsorized Duration (mean +/- se) Winter Storm Uri Removed	Significance
All BWNs	10.9 ± 0.36	11.1 ± 0.40	5.47 ± 0.03	5.17 ± 0.03	
No Weather Event	10.7 ± 0.48	–	5.06 ± 0.04	–	
Heat/Drought	12.8 ± 0.90	12.9 ± 0.93	5.42 ± 0.07	12.9 ± 0.93	
Winter Weather	8.8 ± 0.47	9.9 ± 2.0	7.55 ± 0.07	5.9 ± 0.2	
Hurricane	12.1 ± 2.5	–	7.93 ± 0.33	–	
Tornado	6.7 ± 0.81	–	5.55 ± 0.24	–	
Storm	9.6 ± 1.5	–	4.91 ± 0.11	–	
Flood	8.2 ± 0.80	–	5.91 ± 0.17	–	

Table S4. IRR and Percent Change for Various Fixed-Effects for the Occurrence Model. The value and the 95% confidence interval are shown for both the IRR and percent change. See Section 2.3 BWN Occurrence Model and Equation 1 for details.

	**IRR**								**Percent Change**							
	**OLS**		**FE: Utility**		**FE: Utility x Time**		**FE: Time**		**OLS**		**FE: Utility**		**FE: Utility x Time**		**FE: Time**	
**Event**	**All Data**	**No Uri**	**All Data**	**No Uri**	**All Data**	**No Uri**	**All Data**	**No Uri**	**All Data**	**No Uri**	**All Data**	**No Uri**	**All Data**	**No Uri**	**All Data**	**No Uri**
Heat & Drought	0.955 (0.880, 1.037)	0.92*** (0.89, 0.96)	0.93*** (0.89, 0.97)	1.000 (0.988, 1.013)	0.998 (0.985, 1.010)	0.996 (0.904, 1.097)	0.996 (0.904, 1.097)	-5.8% (-13.2, 2.2%)	-4.5% (-12.0, 3.7%)	-7.7% (-11.5, -3.8%)	-7.1% (-10.9, -3.2%)	+0.00% (-1.24, 1.25%)	-0.25% (-1.47, 0.99%)	-0.41% (-9.58, 9.70%)	-0.41% (-9.58, 9.70%)	0.955 (0.880, 1.037)
Hurricane	1.85*** (1.67, 2.06)	1.76*** (1.64, 1.89)	1.77*** (1.65, 1.90)	1.21*** (1.15, 1.27)	1.21*** (1.15, 1.27)	1.33*** (1.19, 1.49)	1.33*** (1.19, 1.49)	+85.0% (66.6, 105.4%)	+85.3% (66.8, 105.7%)	+76.3% (64.1, 89.5%)	+77.2% (64.9, 90.5%)	+20.5% (14.8, 26.6%)	+21.2% (15.4, 27.4%)	+33.4% (19.5, 48.9%)	+33.4% (19.5, 48.9%)	1.85*** (1.67, 2.06)
Winter Weather	1.16*** (1.09, 1.24)	1.57*** (1.50, 1.64)	1.15*** (1.10, 1.19)	1.45*** (1.40, 1.51)	1.20*** (1.17, 1.23)	1.46*** (1.38, 1.54)	1.46*** (1.38, 1.54)	+57.3% (48.7, 66.3%)	+15.9% (8.6, 23.8%)	+56.8% (49.6, 64.3%)	+14.9% (10.5, 19.5%)	+45.3% (40.0, 50.8%)	+20.0% (16.8, 23.3%)	+45.9% (38.0, 54.3%)	+45.9% (38.0, 54.3%)	1.16*** (1.09, 1.24)
Tornado	1.07* (1.00, 1.14)	1.08*** (1.05, 1.11)	1.08*** (1.05, 1.12)	1.012 (0.996, 1.028)	1.007 (0.991, 1.023)	1.014 (0.953, 1.080)	1.014 (0.953, 1.080)	+6.9% (0.5, 13.7%)	+6.9% (0.5, 13.7%)	+8.0% (4.7, 11.4%)	+8.1% (4.9, 11.5%)	+1.2% (-0.4, 2.8%)	+0.71% (-0.85, 2.30%)	+1.4% (-4.7, 8.0%)	+1.4% (-4.7, 8.0%)	1.07* (1.00, 1.14)
Storm	0.89*** (0.84, 0.94)	0.94*** (0.93, 0.96)	0.95*** (0.93, 0.96)	1.008* (1.000, 1.015)	1.008* (1.001, 1.015)	0.92** (0.86, 0.98)	0.92** (0.86, 0.98)	-11.8% (-16.7, -6.7%)	-11.3% (-16.1, -6.1%)	-5.6% (-7.1, -4.1%)	-5.2% (-6.7, -3.7%)	+0.76% (0.04, 1.49%)	+0.82% (0.11, 1.53%)	-8.3% (-13.8, -2.4%)	-8.3% (-13.8, -2.4%)	0.89*** (0.84, 0.94)
Flood	0.92* (0.86, 0.99)	0.92*** (0.90, 0.95)	0.92*** (0.90, 0.95)	1.031*** (1.017, 1.045)	1.033*** (1.020, 1.047)	1.022 (0.952, 1.096)	1.022 (0.952, 1.096)	-7.8% (-13.8, -1.3%)	-7.6% (-13.6, -1.1%)	-7.9% (-10.4, -5.3%)	-7.6% (-10.2, -5.0%)	+3.1% (1.7, 4.5%)	+3.3% (2.0, 4.7%)	+2.2% (-4.8, 9.6%)	+2.2% (-4.8, 9.6%)	0.92* (0.86, 0.99)

Duration Model Selection

Collinearity matrices and variance inflation factor (VIF) analysis were used to identify and remove any variables creating collinearities in the model. Prior to fixed-effects, all variables were added into an OLS to evaluate collinearity and shows significance in sociodemographic and operational characteristics (Table S5). CWS fixed effects accounted for factors related to the CWS itself that may affect the ability to rescind a BWN and allows for analysis on weather events independent of within CWS variation. Prior to selecting for specific weather events, a simple fixed effect was run to see if the presence of weather events leading up to a BWN had a significant impact on duration. The results indicated a statistically significant ($p < 0.05$) increase of 0.52 days. In addition, week and year fixed effects account for seasonal and temporal trends that may affect BWN duration.

Table S5. OLS Duration Model with All Sociodemographic and Operational Variables. Values show estimate and standard error. * indicate a significant variable.

	OLS
Constant	6.88 ± 0.18 **
Heat & Drought	0.17 ± 0.07 *
Winter Weather	2.69 ± 0.09 ***
Hurricane	2.48 ± 0.26 ***
Storm	-0.34 ± 0.13 **
Tornado	0.19 ± 0.25
Flood	0.63 ± 0.17 ***
Racial Distribution	0.002 ± 0.002
Housing Density	-0.0012 ± 0.00012 ***
Median Income	-0.00002 ± 0.0 ***
Publicly Owned	-0.74 ± 0.07 ***
Groundwater Sourced	0.30 ± 0.06 ***
CWS Size	0.0 ± 0.0 *

Table S6. BWN Duration Model Comparison with Varying Fixed Effects for Unbalanced Panel Data. Values show the estimates and the 95% confidence intervals determined through bootstrap analysis (n = 1000). OLS had no fixed effects, Utility x Time had WSID-year and week fixed effects, Utility had WSID fixed effects, and Time had year and week fixed effects. See Section 2.4 BWN Duration Model and Equation 2 for details.

	All BWNs Included				Without Winter Storm Uri			
	OLS	FE: Utility	FE: Utility x Time	FE: Time	OLS	FE: Utility	FE: Utility x Time	FE: Time
Constant	5.1***				5.1***			
Heat and Drought	0.21* (0.01, 0.40)	0.17* (0.02, 0.34)	0.15 (-0.08, 0.39)	0.12 (-0.06, 0.32)	0.27** (0.07, 0.47)	0.21** (0.06, 0.35)	0.14 (-0.08, 0.36)	0.15 (-0.07, 0.35)
Winter Weather	2.50*** (2.33, 2.68)	2.73*** (2.55, 2.93)	1.60*** (1.15, 2.03)	1.40*** (1.03, 1.76)	0.83*** (0.42, 1.24)	0.61** (0.24, 0.97)	0.07 (-0.48, 0.62)	0.65** (0.21, 1.10)
Hurricane	2.64*** (1.94, 3.38)	2.42*** (1.79, 3.08)	1.10* (0.00, 2.23)	2.49*** (1.72, 3.20)	2.66*** (1.98, 3.34)	2.32*** (1.66, 3.02)	2.20** (0.81, 3.52)	2.56*** (1.86, 3.24)
Storm	0.08 (-0.41, 0.55)	0.12 (-0.32, 0.57)	-0.12 (-0.79, 0.61)	0.11 (-0.39, 0.59)	0.07 (-0.39, 0.55)	0.06 (-0.37, 0.53)	-0.25 (-0.94, 0.45)	0.08 (-0.40, 0.58)
Tornado	-0.34** (-0.57, -0.10)	-0.25* (-0.48, -0.03)	-0.10 (-0.41, 0.19)	-0.36** (-0.62, -0.12)	-0.32* (-0.58, -0.06)	-0.16 (-0.39, 0.06)	-0.06 (-0.35, 0.24)	-0.35** (-0.59, -0.11)
Flood	0.58** (0.23, 0.93)	0.57*** (0.28, 0.86)	0.29 (-0.13, 0.67)	0.59** (0.23, 0.96)	0.60*** (0.24, 0.95)	0.47** (0.16, 0.78)	0.22 (-0.18, 0.61)	0.58*** (0.25, 0.92)
Observations	18,276	18,276	18,276	18,276	16,301	16,301	16,301	16,301
RMSE	3.78	3.00	2.18	3.76	3.84	3.03	2.22	3.81
Adj. R2	0.05	0.28	0.38	0.06	0.01	0.27	0.38	0.02
Within R2	-	0.05	0.08	0.01	--	0.01	0.002	0.01

Table S7. Duration model interaction results for BWN Cause for each weather event. * indicate that the estimate was significant (p < 0.05). See Section 2.4 BWN Duration Model and Equation 2 for details. Section 2.5 Sensitivity Analyses describes the interaction terms.

Weather Event	LDP	LDR	Microbial	Other	Turbidity	Water Outage
Heat & Drought	0.03<br>(-0.19, 0.24)	-1.35<br>(-2.92, 0.21)	0.57<br>(-2.26, 3.49)	0.02<br>(-0.41, 0.47)	3.55<br>(-4.16, 9.83)	0.47**<br>(0.19, 0.78)
Winter Weather	1.78***<br>(1.39, 2.18)	5.36<br>(-0.95, 40.90)	--	0.16<br>(-0.59, 1.02)	0.88<br>(-1.77, 3.29)	-0.11<br>(-1.21, 1.04)
Hurricane	2.42**<br>(0.67, 4.14)	16.94<br>(-7.33, 59.02)	--	0.48<br>(-0.52, 1.57)	--	1.15<br>(-1.70, 3.20)
Tornado	-0.66<br>(-1.32, 0.00)	-5.12<br>(-7.87, 7.99)	--	-0.41<br>(-1.77, 0.80)	--	1.02<br>(-0.22, 2.14)
Storm	0.03<br>(-0.28, 0.31)	-0.72<br>(-2.74, 0.43)	-1.25<br>(-3.45, 0.81)	0.21<br>(-0.44, 0.87)	-9.95<br>(-26.10, 3.54)	-0.18<br>(-0.62, 0.23)
Flood	-0.19<br>(-0.58, 0.19)	4.18<br>(-1.17, 9.62)	14.89***<br>(6.24, 15.84)	2.08***<br>(1.21, 2.98)	5.56<br>(-9.80, 12.37)	-0.18<br>(-0.74, 0.37)

Table S8. Duration model interaction results for CWS Size for each weather event. * indicate that the estimate was significant (p < 0.05). See Section 2.4 BWN Duration Model and Equation 2 for details. Section 2.5 Sensitivity Analyses describes the interaction terms.

Event	Small	Medium/Large
Heat & Drought	0.10<br>(-0.13, 0.35)	0.24<br>(-0.03, 0.52)
Winter Weather	1.92***<br>(1.53, 2.36)	0.86***<br>(0.44, 1.31)
Hurricane	1.14*<br>(0.23, 2.12)	1.19<br>(-2.35, 3.87)
Tornado	0.39<br>(-0.31, 1.08)	-1.14**<br>(-1.96, -0.24)
Storm	-0.34*<br>(-0.67, -0.04)	0.26<br>(-0.06, 0.59)
Flood	0.33<br>(-0.12, 0.75)	0.21<br>(-0.24, 0.69)

Table S9. Duration model interaction results for CWS Owner for each weather event. * indicate that the estimate was significant ($p < 0.05$). See Section 2.4 BWN Duration Model and Equation 2 for details. Section 2.5 Sensitivity Analyses describes the interaction terms.

Event	Public	Private
Heat & Drought	0.07<br>(-0.12, 0.27)	0.50*<br>(0.07, 0.93)
Winter Weather	1.01***<br>(0.65, 1.38)	2.73***<br>(2.23, 3.28)
Hurricane	1.91**<br>(0.50, 3.23)	1.11*<br>(0.05, 2.21)
Tornado	-0.52<br>(-1.14, 0.13)	0.64<br>(-0.28, 1.53)
Storm	-0.05<br>(-0.29, 0.20)	-0.22<br>(-0.68, 0.25)
Flood	0.10<br>(-0.27, 0.49)	0.59<br>(-0.03, 1.17)

Table S10. Duration model interaction results for CWS Source for each weather event. * indicate that the estimate was significant ($p < 0.05$). See Section 2.4 BWN Duration Model and Equation 2 for details. Section 2.5 Sensitivity Analyses describes the interaction terms.

Event	Groundwater	Surface Water
Heat & Drought	0.26*<br>(0.00, 0.50)	0.04<br>(-0.24, 0.31)
Winter Weather	2.04***<br>(1.60, 2.50)	1.02***<br>(0.62, 1.42)
Hurricane	0.97*<br>(0.08, 1.87)	3.77<br>(-0.80, 8.15)
Tornado	0.25<br>(-0.46, 0.95)	-0.72<br>(-1.65, 0.16)
Storm	-0.17<br>(-0.47, 0.13)	0.01<br>(-0.31, 0.31)
Flood	0.33<br>(-0.09, 0.73)	0.15<br>(-0.35, 0.63)

Table S11. Duration model interaction results for Housing Density for each weather event. * indicate that the estimate was significant ($p < 0.05$). See Section 2.4 BWN Duration Model and Equation 2 for details. Sectio 2.5 Sensitivity Analyses describes the interaction terms.

Event	Rural	Urban
Heat & Drought	-0.00<br>(-0.22, 0.21)	0.46**<br>(0.13, 0.77)
Winter Weather	1.48***<br>(1.08, 1.89)	1.75***<br>(1.30, 2.19)
Hurricane	1.29<br>(-0.25, 3.09)	1.03<br>(-0.06, 2.09)
Tornado	-0.53<br>(-1.20, 0.14)	0.46<br>(-0.45, 1.39)
Storm	-0.17<br>(-0.48, 0.13)	-0.04<br>(-0.37, 0.28)
Flood	0.15<br>(-0.33, 0.63)	0.39<br>(-0.04, 0.83)

Table S12. Duration model interaction results for Income Class for each weather event. * indicate that the estimate was significant ($p < 0.05$). See Section 2.4 BWN Duration Model and Equation 2 for details. Section 2.5 Sensitivity Analyses describes the interaction terms.

Weather Event	High Income	Middle Income	Low Income
Heat & Drought	0.50**<br>(0.12, 0.84)	0.02<br>(-0.18, 0.22)	0.33<br>(-1.74, 2.12)
Winter Weather	1.94***<br>(1.48, 2.37)	1.48***<br>(1.08, 1.91)	-1.09<br>(-2.84, 0.79)
Hurricane	0.96<br>(-0.00, 1.94)	1.39<br>(-0.21, 3.13)	--
Tornado	0.40<br>(-0.47, 1.26)	-0.47<br>(-1.15, 0.21)	--
Storm	-0.15<br>(-0.55, 0.26)	-0.05<br>(-0.33, 0.21)	-0.85<br>(-2.12, 0.30)
Flood	0.05<br>(-0.40, 0.49)	0.40*<br>(0.01, 0.84)	0.54<br>(-6.03, 9.11)

Table S13. Duration model interaction results for Racial Distribution for each weather event. * indicate that the estimate was significant ($p < 0.05$). See Section 2.4 BWN Duration Model and Equation 2 for details. Section 2.5 Sensitivity Analyses describes the interaction terms.

**Weather Event**	**White**	**POC**
Heat & Drought	0.14<br>(-0.06, 0.35)	0.23<br>(-0.09, 0.56)
Winter Weather	1.60***<br>(1.24, 1.99)	1.42***<br>(0.90, 1.97)
Hurricane	4.08***<br>(2.76, 5.57)	-0.39<br>(-1.39, 0.77)
Tornado	-0.18<br>(-0.85, 0.51)	0.42<br>(-0.37, 1.22)
Storm	-0.05<br>(-0.33, 0.20)	-0.28<br>(-0.68, 0.13)
Flood	0.15<br>(-0.25, 0.56)	0.31<br>(-0.21, 0.83)

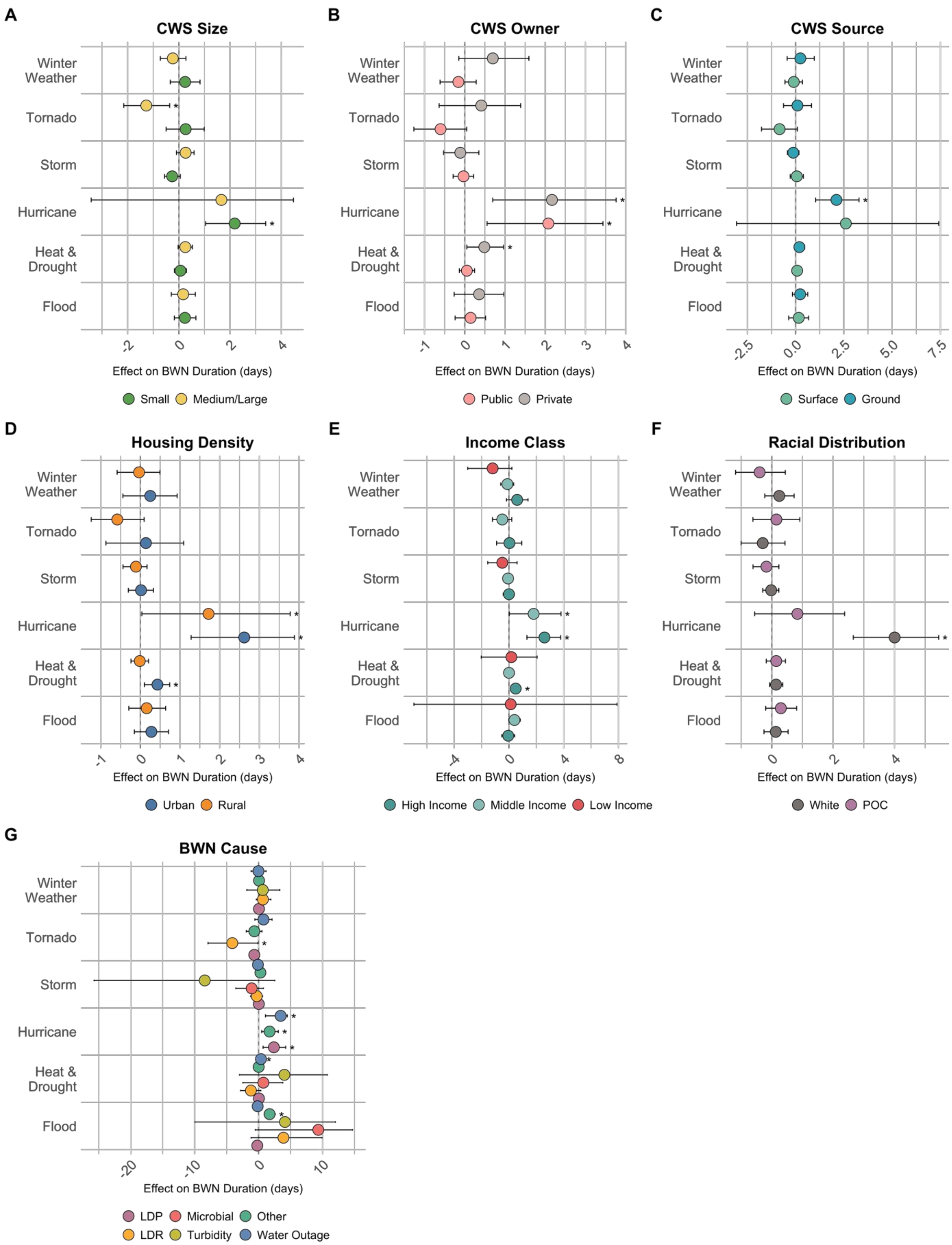

Figure S3. Duration Model Interaction Results Excluding Winter Storm Uri.

Bottled Water Sales

Based on the dataset provided, urban centers accounted for the majority of total sales (Figure S1), but on a per capita basis, other counties had the highest bottled water sales (Figure S2). Culberson County in Western Texas had the highest bottled water sales per capita ($822), followed by counties in the northeast: Rains ($185), Marion ($180), Camp ($173), and Gregg ($169). For many counties in West Texas and the panhandle area, there was limited data available.

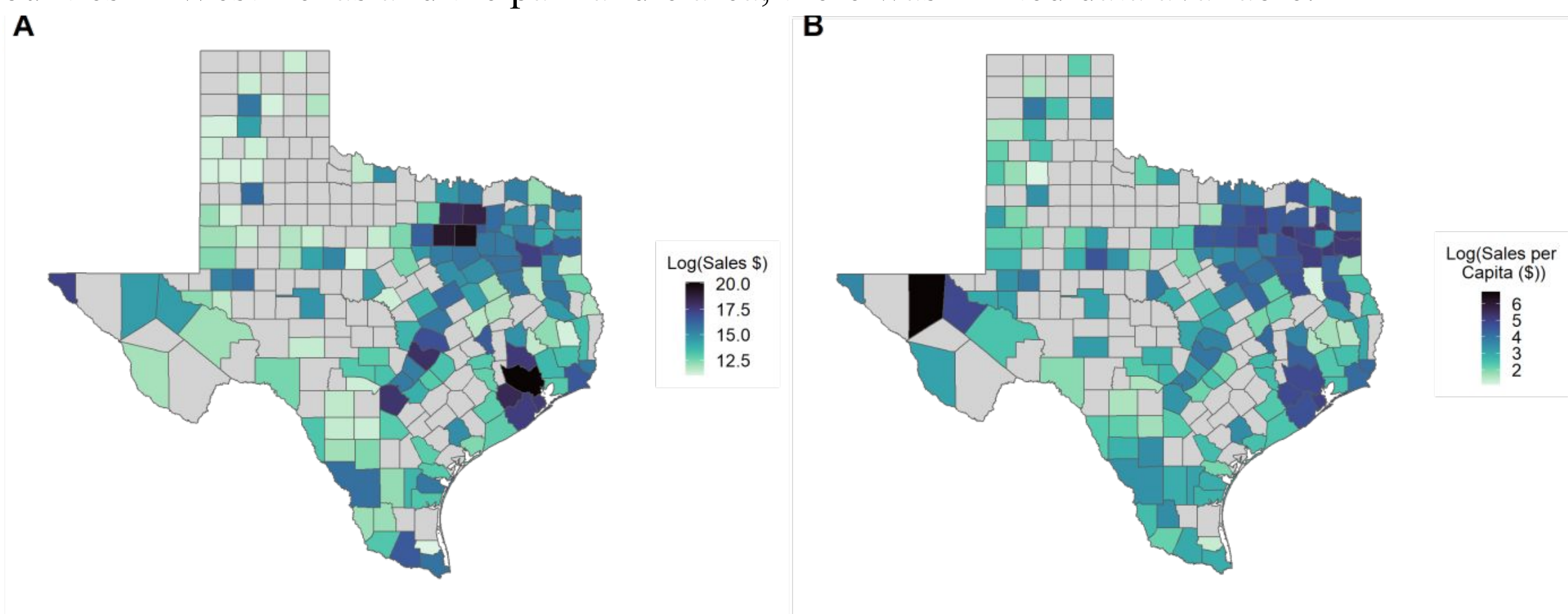

Figure S4. Total Sales and Total Sales per Capita at the County Level in Texas. A) Total sales in each county there is data for across the whole dataset. B) Total per capita sales were calculated by taking the mean county population and dividing the total sales for the county.

Table S14. Bottled Water Sales Analysis for Weather Events and BWN. Estimates are based on bootstrapping with 1000 reps using county-by-year and week fixed effects. A dummy variable was created which was 1 if there was a weather event and 0 if there was no weather event.

	**Coefficient Estimate**
Weather Event	-0.0023<br>(-0.0065, 0.002)
BWN Only	0.057***<br>(0.0049, 0.11)
BWN + Weather	0.11<br>(0.044, 0.18)

Table S15. Mean Baseline Bottled Water Sales in Majority POC vs. Majority White Counties. The p-value is based on a t-test.

	POC Counties	White Counties	P-value
Sales ($)	38,809.07	13,920.12	0.20
Per Capita Sales ($/person)	0.074	0.085	0.68
Sales (oz)	3,242,741	1,259,193	0.2
Per Capita Sales (oz/person)	4.48	7.45	0.02

Note: NeilsonIQ does not consistently provide data on volume of water sold, so analyses are shown with dollars.

Bottled Water Sales Model Selection

Exclusions to the data set were evaluated at the county and county and store level. A Hausman test was conducted to evaluate the suitability of fixed effects or random effects models, which resulted in the fixed effects model being selected ($p < 0.05$). For the selected model, collinear variables were removed and a year and week fixed effect was included. Lag variables were created to analyze the effect of the previous week's weather on BWN indicators and bottled water sales. The lag variables had no significant impact on bottled water sales ($p = 0.98$), so they were excluded from the final model.

Table S16. Model Results for Bottled Water Model. Each box shows the estimate and the 95% confidence interval from bootstrapping (n = 1000). See Section 2.5 Bottled Water Sales Model and Equation 3 for details.

	OLS	FE: County	FE: County x Time	FE: Time
Constant	7.51			
BWN Indicator	0.77<br>(-0.56, 2.17)	0.29***<br>(0.15, 0.42)	0.069**<br>(0.019, 0.12)	0.55<br>(-0.64, 1.67)
Heat & Drought	-0.21*<br>(-0.39, -0.03)	-0.035**<br>(-0.056, -0.013)	0.015***<br>(0.0083, 0.022)	-0.23*<br>(-0.42, -0.040)
Winter Weather	-0.41***<br>(-0.57, -0.24)	-0.24***<br>(-0.26, -0.22)	-0.026***<br>(-0.037, -0.014)	-0.23**<br>(-0.41, -0.090)
Hurricane	0.46<br>(-0.14, 1.11)	0.32***<br>(0.24, 0.40)	0.16***<br>(0.099, 0.23)	0.40<br>(-0.32, 0.80)
Tornado	0.39**<br>(0.18, 0.59)	0.0096<br>(-0.013, 0.031)	-0.0061<br>(-0.020, 0.0077)	0.41***<br>(0.20, 0.61)
Flood	0.45***<br>(0.36, 0.54)	0.032***<br>(0.021, 0.043)	-0.011***<br>(-0.016, -0.0062)	0.44***<br>(0.35, 0.54)
Storm	0.88***<br>(0.74, 1.03)	0.0071<br>(-0.0063, 0.022)	-0.056***<br>(-0.065, -0.046)	0.87***<br>(0.72, 1.00)
Observations	98,556	98,556	98,556	98,556
RMSE	2.10	0.85	0.21	2.02
Adj. R2	0.02	0.84	0.99	0.09
Within R2	--	0.11	0.004	0.01

Table S17. Bottled water sales interaction results for Housing Density for each weather event. * indicate that the estimate was significant (p < 0.05). The estimate shows the percent change in bottled water sales. See Section 2.5 Bottled Water Sales Model and Equation 3 for details. Section 2.5 Sensitivity Analyses describes the interaction terms.

**Event**	**Rural**	**Urban**
BWN Indicator	0.0648*<br>(0.0148, 0.1236)	0.0839*<br>(0.0241, 0.1552)
Heat & Drought	0.0182***<br>(0.0117, 0.0244)	0.0052<br>(-0.0018, 0.0119)
Winter Weather	-0.0366***<br>(-0.0467, -0.0257)	0.0185***<br>(0.0089, 0.0281)
Hurricane	0.1456***<br>(0.0769, 0.2174)	0.1928***<br>(0.1298, 0.2734)
Tornado	-0.0160*<br>(-0.0296, -0.0020)	0.0156<br>(-0.0001, 0.0295)
Storm	-0.0038<br>(-0.0089, 0.0011)	-0.0296***<br>(-0.0339, -0.0251)
Flood	-0.0595***<br>(-0.0701, -0.0484)	-0.0478***<br>(-0.0581, -0.0376)

Table S18. Bottled water sales interaction results for Income Class for each weather event. * indicate that the estimate was significant ($p < 0.05$). The estimate shows the model coefficient in bottled water sales. See Section 2.5 Bottled Water Sales Model and Equation 3 for details. Section 2.5 Sensitivity Analyses describes the interaction terms.

Event	High Income	Middle Income	Low Income
BWN Indicator	0.2689** (0.0861, 0.4716)	0.0657** (0.0285, 0.1130)	-0.0557 (-0.1868, 0.1174)
Heat & Drought	0.0088 (-0.0004, 0.0175)	0.0154*** (0.0088, 0.0219)	0.0292** (0.0076, 0.0486)
Winter Weather	-0.0101 (-0.0253, 0.0074)	-0.0239*** (-0.0347, -0.0126)	-0.0625*** (-0.0899, -0.0339)
Hurricane	0.2157*** (0.1699, 0.2639)	0.1450*** (0.0781, 0.2214)	0.1411 (-0.1950, 0.3542)
Tornado	0.0196* (0.0007, 0.0360)	-0.0154* (-0.0290, -0.0019)	-0.0274 (-0.0962, 0.0285)
Storm	-0.0257*** (-0.0319, -0.0197)	-0.0067* (-0.0121, -0.0017)	0.0017 (-0.0137, 0.0170)
Flood	-0.0540*** (-0.0677, -0.0395)	-0.0574*** (-0.0665, -0.0479)	-0.0530*** (-0.0791, -0.0292)

Table S19. Bottled water sales interaction results for Racial Distribution for each weather event. * indicate that the estimate was significant ($p < 0.05$). The estimate shows the model coefficient in bottled water sales. See Section 2.5 Bottled Water Sales Model and Equation 3 for details. Section 2.5 Sensitivity Analyses describes the interaction terms.

Event	White	POC
BWN Indicator	0.0641**<br>(0.0214, 0.1110)	0.0791*<br>(0.0105, 0.1636)
Heat & Drought	0.0080**<br>(0.0028, 0.0133)	0.0294***<br>(0.0181, 0.0405)
Winter Weather	-0.0037<br>(-0.0143, 0.0075)	-0.0560***<br>(-0.0697, -0.0409)
Hurricane	0.2043***<br>(0.1392, 0.2711)	0.1473***<br>(0.0842, 0.2245)
Tornado	-0.0015<br>(-0.0135, 0.0099)	-0.0119<br>(-0.0326, 0.0102)
Storm	-0.0145***<br>(-0.0190, -0.0103)	-0.0056<br>(-0.0125, 0.0009)
Flood	-0.0481***<br>(-0.0582, -0.0372)	-0.0657***<br>(-0.0764, -0.0550)
